# ICU predictive factors of fibrotic changes following COVID-19 related ARDS: a RECOVIDS substudy

**DOI:** 10.1186/s13613-025-01577-2

**Published:** 2025-11-04

**Authors:** Matthieu Demeyere, Isabelle Fournel, Amadou-Khalilou Sow, Stéphanie Gélinotte, Martine Nyunga, Anissa Berraies, Marie Labruyère, Alexandre Ampere, Bertrand Sauneuf, Cédric Daubin, Agathe Delbove, Julio Badie, Pierre Bulpa, David Delacour, Clotilde Lefevre, Saad Nseir, Elise Artaud-Macari, Michel Ramakers, Vanessa Bironneau, Hugues Georges, Walid Oulehri, Arnaud-Felix Miailhe, Nicolas Delberghe, Béatrice La Combe, Elise Redureau, Caroline Clarot, Nicholas Sedillot, Thierry Dugernier, David Schnell, Laurie Lagache, Charlotte Salmon Gandonniere, Julien Maizel, Thierry Vanderlinden, Gaël Bourdin, Mélanie Adda, Gaëtan Plantefeve, Gaëtan Beduneau, Marjolaine Georges, Jean-Pierre Quenot, Pierre-Louis Declercq, RECOVIDS Study Group and the CRICS-TRIGGERSEP and BOREAL Research Networks

**Affiliations:** 1 Service de Radiologie, CHU Rouen, 76000 Rouen, France; 2 CHU Dijon Bourgogne, INSERM, Université de Bourgogne, CIC 1432, Module Epidémiologique Clinique, 21000 Dijon, France; 3 Service de Médecine Intensive Réanimation, Centre Hospitalier de Dieppe, 76200 Dieppe, France; 4 Service de Médecine Intensive Réanimation, CH de Roubaix, Roubaix, France; 5 Service de Pneumologie, CH Chartres, Chartres, France; 6 Department of Intensive Care, Burgundy University Hospital, Dijon, France; 7 Lipness Team, INSERM Research Center LNC-UMR1231 and LabEx LipSTIC, University of Burgundy, Dijon, France; 8 INSERM CIC 1432, Clinical Epidemiology, University of Burgundy, Dijon, France; 9 Service de Pneumologie, CH de Béthune, Béthune, France; 10 Service de Médecine Intensive Réanimation, CH Public du Cotentin, Cherbourg-en-Cotentin, France; 11 Department of Medical Intensive Care, CHU de Caen Normandie, Caen, France; 12 Service de Réanimation Polyvalente, CHBA Vannes, Vannes, France; 13 Service de Médecine Intensive Réanimation, Hopital Nord Franche-Comte, Trevenans, France; 14 Service des Soins Intensifs, Mont-Godinne University Hospital, CHU UCL Namur, Yvoir, Belgium; 15 Service de Radiologie, Clinique du Cèdre, Bois-Guillaume, France; 16 Service de Médecine Intensive Réanimation, CHU de Lille, Lille, France; 17 Inserm U1285, Univ. Lille, CNRS, UMR 8576-UGSF-Unité de Glycobiologie Structurale et Fonctionnelle, Lille, France; 18 Department of Pneumology, Thoracic Oncology and Respiratory Intensive Care Unit, Normandie Univ, UNIROUEN, EA3830, CHU Rouen, Rouen, France; 19 Service de Médecine Intensive Réanimation, Centre Hospitalier Mémorial de Saint-Lô, Saint-Lô, France; 20 Service de Pneumologie, CHU Poitiers, INSERM CIC 1402, ALIVES Research Group, Université de Poitiers, Poitiers, France; 21 Service de Médecine Intensive Réanimation, CH de Tourcoing, Tourcoing, France; 22 Service de Réanimation Chirurgicale, CHRU Strasbourg, Strasbourg, France; 23 Service de Médecine Intensive Réanimation, CHU Nantes, Nantes, France; 24 Department of Intensive Care Medicine, St James Hospital, Dublin, Ireland; 25 Service de Pneumologie, CHES Evreux, Evreux, France; 26 Service de Réanimation Polyvalente, Groupe Hospitalier Bretagne Sud, Lorient, France; 27 Service de Pneumologie, CHD Vendée, Vendée, France; 28 Service de Pneumologie, CH d’Abbeville, Abbeville, France; 29 Service de Réanimation Polyvalente, Centre Hospitalier Fleyriat, Bourg en Bresse, France; 30 Service de Soins Intensifs, Clinique Saint Pierre, 1340 Ottignies, Belgique; 31 Service de Réanimation Polyvalente et USC, CH d’Angoulême, d’Angoulême, France; 32 Service de Réanimation Médico-Chirugicale, Groupe Hospitalier du Havre, Le Havre, France; 33 Service de Médecine Intensive Réanimation, CHRU de Tours, INSERM CIC 1415, CRICS-TriGGERSep., Tours, France; 34 Service de Médecine Intensive Réanimation, CHU d’Amiens, d’Amiens, France; 35 Intensive Care Unit, St Philibert Hospital, ETHICS EA 7446, Lille Catholic University, Lille, France; 36 Service de Réanimation Polyvalente, Hôpital Saint Joseph Saint Luc, Lyon, France; 37 Service de Médecine Intensive Réanimation, Hôpitaux de Marseille, Hôpital Nord, Marseille, France; 38 Service de Médecine Intensive Réanimation, CH d’Argenteuil, d’Argenteuil, France; 39 Department of Medical Intensive Care, Univ Rouen Normandie, GRHVN UR 3830, CHU Rouen, 76000 Rouen, France; 40 Department of Pulmonary Medicine and Intensive Care Unit, University Hospital, Dijon, France

**Keywords:** ARDS, COVID-19, Fibrosis, Computed tomography, Risk factors

## Abstract

**Background:**

Pulmonary fibrotic changes (FC) following COVID-19-related ARDS represent a significant concern due to the potential respiratory complications. The identification of early predictive factors for FC and the development of predictive tools are needed to optimize patient management and outcomes.

**Methods:**

This observational prospective multicentre study is a substudy of the RECOVIDS study and included 32 centres in France and Belgium. COVID-19 ARDS survivors were included if they met the Berlin ARDS criteria or if they received high flow oxygen therapy (flow ≥ 50 L/min and FiO_2_ ≥ 50%). Exclusion criteria were non-attendance at follow-up 6 ± 1 months after ICU discharge, lack of baseline or follow-up chest CT, and history of interstitial lung disease. The primary endpoint was presence of FC at follow-up CT. The secondary outcome was to identify predominant radiological patterns.

**Results:**

Among 555 patients included in the RECOVIDS study, 440 were analysed, of whom 162 (36.8%) had FC at follow-up. Predictive factors for FC included older age, body mass index < 30, Charlson comorbidity index ≥ 1, invasive mechanical ventilation, early signs of FC, and greater lung involvement on baseline CT. The nomogram for predicting pulmonary FC yielded an AUC of 80.6% (95%CI (76.4–84.8)). Late organizing pneumonia was the most common pattern overall and 30 (18.5%) of the 162 patients with FC presented mainly anterior fibrosis compatible with post ventilatory changes.

**Conclusion:**

In this large cohort of COVID-19 ARDS survivors, 36.8% exhibited FC at 6 months post-ICU discharge. The key predictors identified here could guide therapeutic and follow-up strategies.

**Supplementary Information:**

The online version contains supplementary material available at 10.1186/s13613-025-01577-2.

## Introduction

The SARS-CoV-2 pandemic, necessitate the exploration and management of late consequences of acute infection, particularly respiratory sequelae. Among the most concerning is pulmonary fibrosis, which can develop following viral infection [1], and carries a risk of clinical impact, including respiratory disability and impaired quality of life (QoL), somewhat similar to idiopathic pulmonary fibrosis (IPF) [2], The possibility of fibrosing lung injury post-COVID-19 has been documented [3], yet the course of such injury remains unclear. Its occurrence appears to be associated with age and the severity of the initial respiratory insult, especially in case of acute respiratory distress syndrome (ARDS) [4]. The pulmonary and systemic inflammatory response following SARS-CoV-2 aggression is more pronounced in patients who develop ARDS potentially sustaining an inflammatory and/or autoimmune process that could lead to interstitial lung lesions [5]. Additionally, ARDS fibrotic sequelae were described long before COVID-19 era [6]. Acute COVID-19 infection and IPF share common cellular and molecular features, raising concerns about a similar progressive potential in some patients [7]. Early identification in the intensive care unit (ICU) of patients at highest risk of fibrotic changes (FC) could help optimize their management and follow-up. We therefore aimed to identify predictors of post-COVID-19 pulmonary fibrosis, based on data from the initial chest computed tomography (CT) and ICU stay in a cohort of COVID-19 ARDS survivors.

## Methods

### Study design

This prospective observational multicentre study is a substudy of the RECOVIDS study, performed in 30 centres in France and 2 in Belgium. The protocol and results of the main study have previously been published [8, 9]. The RECOVIDS study was approved by the ethics committee “Comité de Protection des Personnes Sud Méditerranée II” on 10/07/2020 under the number 2020-A02014-35 for France, and by the ethics committee of Namur on 11/12/2020 under the number B0392020000073 for Belgium. Oral informed consent was obtained from all patients.

All adult patients admitted to ICU due to SARS-CoV-2 infection confirmed by polymerase chain reaction, in any of the participating centres, were eligible. To be included, patients had to have undergone baseline CT at the initial phase of management; have ARDS diagnosed according to the Berlin 2012 definition [10] or have received high flow nasal oxygen (HFOT) with a flow of at least 50L/minute with FiO_2_ > 50% and a PaO_2_/FiO_2_ ratio ≤ 200. The non-inclusion criteria of the main study [9] are presented in the additional file (eMethods 1).

Specific exclusion criteria for this substudy were: failure to attend follow-up CT, or a history of interstitial lung disease (ILD). Additionally, patients in whom baseline CT was performed more than 21 days after ICU admission, or in whom the follow-up CT showed FC but was done less than 4 months after baseline CT, or patients with CT scan of insufficient quality to assess the primary outcome were excluded from the statistical analysis as protocol violations.

### Procedures

Patients were screened at ICU discharge and during hospitalization in post-ICU units. The follow-up, including a follow-up CT, was scheduled at 6 ± 1 months after ICU discharge as previously described [9].

Following anonymization, radiological data were transferred to the coordinating centre on a secure server for central interpretation.

### Outcomes and chest CT analysis

The primary outcome was presence of FC, defined as interstitial lesions with volume loss and/or architectural distortion and/or traction bronchiectasis, and/or honeycombing at follow-up CT. FC at baseline, termed later in the text “early fibrotic signs” and follow-up, termed “FC”, were independently assessed by two thoracic radiologists, both actively involved in ILD multidisciplinary meetings. Discrepancies were collaboratively resolved during a concurrent third review session. For the purposes of the present study, radiological features on baseline CT at the acute phase were described using the same terminology applied in fibrotic ILD and follow-up CT evaluation, allowing systematic tracking of changes. However, the authors acknowledge that the use of fibrotic descriptors—particularly traction bronchiectasis—in the acute setting should be avoided as such features may be reversible and not indicative of established fibrosis. Moreover seemingly pre-existing subpleural fibrotic interstitial lung abnormalities on baseline CT were not considered as FC on baseline [11], or at follow-up, unless fibrotic progression was observed after reviewing both CTs. Quality was rated as follows: 1-no artefact; 2-mild artefact; 3-moderate artefact (< 10%), 4-severe artefact (> 10%), 5-any artefact precluding a conclusion as to the presence of FC. CT scans rated 5 were excluded from the statistical analysis.

The main secondary outcomes of radiological signs rated at baseline and follow-up CT are listed in additional file (eMethods 2). The extent of lesions was quantified using a visual method through an average score (0–100%) and an automated method using the CT Pneumonia Analysis application on Syngo.Via (Siemens Healthineers, Erlangen, Germany), see additional file (eMethods 3).

The main pattern on follow-up CT was categorized as one of the following: complete resolution (confirmed using minimal intensity projection), minimal lesions < 5% of any area (isolated reticulation, pleural bands), residual ground glass opacities (GGO), late organizing pneumonia (OP) with or without distortion, anterior fibrosis, and any other types of fibrotic pattern [12].

The non-radiological secondary outcomes were the clinical and functional respiratory function, assessed using the modified Medical Research Council dyspnea scale (mMRC) [13], pulmonary function tests (PFT) and the 6-min walk test (6MWT) [14]. Quality of life (QoL) was evaluated using the Visual Simplified Respiratory Questionnaire (VRSQ) [15] and Short Form Health Survey modified 36 (SF-36) [16].

### Statistical analysis

All analyses were performed using R (version 4.3.0; R Core Team, 2023). After assessing normality using the Shapiro–Wilk test, continuous variables were summarized as means with standard deviation (SD) or as medians with interquartile ranges (IQR) for normally and non-normally distribution respectively. Group differences for continuous variables were tested using Student’s t-test for normally distributed data and the Wilcoxon test for non-normally distributed data. Categorical variables are presented as counts and proportions, and were compared using the Fisher exact or Chi-square test, as appropriate. Lung involvement was presented as mean percentage of the total lung or pulmonary region, considered as continuous variables and expressed as median (IQR). Missing data were reported in the tables, by reporting subjects with available data as [N0] but excluded from statistical analyses.

Variables associated with FC at follow-up were identified using the strategy below. Initially, candidate variables (additional file, Table S1) with a p-value < 0.15 by univariable analysis were included in a multivariable logistic regression model, excluding those with few events or redundancy (full model). A comparison of area under the curve (AUC) was performed to evaluate the diagnostic performance of automated versus visual quantification of lung involvement on baseline CT. The best variable was chosen for inclusion as the lung extent parameter in the multivariable model (eFigure 1).

Collinearity between adjustment variables was verified by calculating the variance inflation factor (VIF); all values were < 5, indicating absence of multicollinearity. Backward stepwise selection was applied to obtain final model, with model improvements assessed by the Akaike Information Criterion (AIC).

The Box-Tidwell test was used to check the non-linearity of continuous variables. Model calibration was assessed with a visual calibration plot and the Hosmer–Lemeshow test. Results are presented as odds ratios (ORs) and 95% confidence intervals (CIs).

Internal validation of the model was performed using cross-validation: the population was randomly split into a 80% training set and a 20% test set [17]. Model performance was evaluated using the area under the curve (AUC) metric, ranging from 50% (no discrimination) to 100% (perfect discrimination).

A nomogram based on the final model was constructed using the entire dataset [18]. Internal validation of the nomogram's predictive accuracy was conducted using 1000 bootstrap replications [18].

## Results

### Study population

**Fig. 1:**
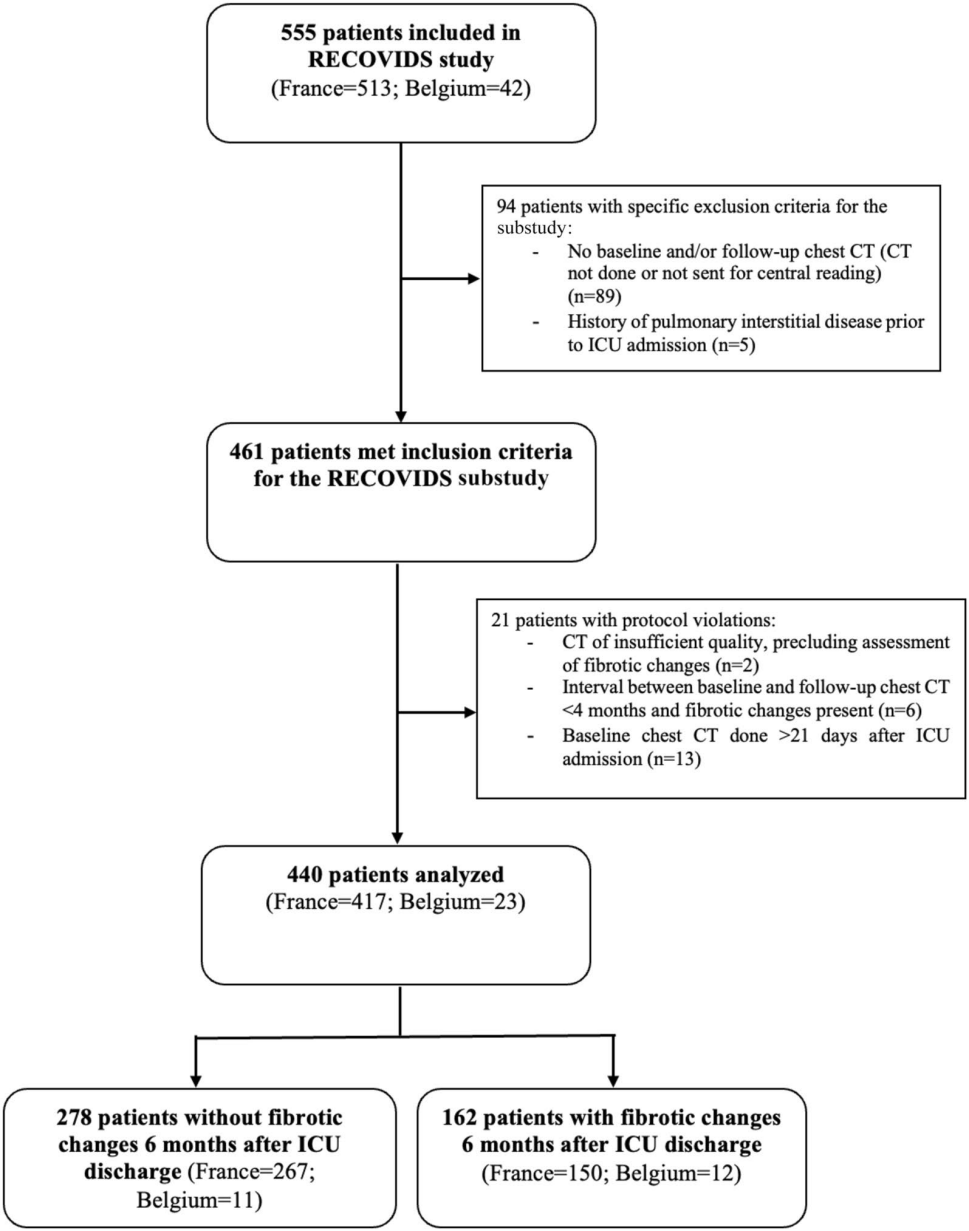
Flow chart of the study population

The RECOVIDS study included a total of 555 adult patients between September 2020 and June 2021 (513 in France, 42 in Belgium). Among these, 94 had specific exclusion criteria for the present substudy. An additional 21 patients had protocol violations, leaving a total of 440 patients for the present analysis (Fig. 1).

Of these 440 patients, 162 (36.8%) had FC at follow-up. These patients were significantly older, more often men, less often obese, more frequently with Charlson score ≥ 1, and had higher median Simplified Acute Physiology Score II (SAPS II), compared to patients without FC (Table 1). Patients with FC at follow-up more often had early fibrotic signs at baseline (46% vs 29%; p < 0.001), as well as more extensive lung involvement, as assessed by visual analysis (51.8% [38.9–69.8] vs 37.5% [23.7–52.9]); p < 0.001) or with CT Pneumonia analysis (57.3% [41.7–69.8] vs 44.3 [28.8, 61.1]); p < 0.001) (Table 2). Detailed lung involvement by pulmonary region, or according to the interval between the initial CT scan and ICU admission in the FC group is described in the additional file (Table S2 and Fig. S2 respectively).

**Table 1:** Patient characteristics and management at admission and during ICU stay according to fibrotic changes

	All patients, N = 440	No fibrotic changes N = 278 (63.2)	Fibrotic changes N = 162 (36.8)	*P*-value
Age, mean (SD)	63.2 (11)	61.2 (11.8)	66.7 (8.6)	< 0.001
Sexe, female	134 (30.5%)	97 (34.9%)	37 (22.8%)	< 0.008
COVID-19 epidemic wave				0.362
First wave	148 (33.6%)	87 (31.3%)	61 (37.7%)	
Second wave	239 (54.3%)	155 (55.8%)	84 (51.9%)	
Third wave	53 (12.1%)	36 (12.9%)	17 (10.5%)	
Pre‐existing conditions				
Alcohol				0.653
Never or former	178/399 (44.6%)	111/242 (45.9%)	67/157 (42.7%)	
Up to one drink per day	172/399 (43.1%)	104/242 (43%)	68/157 (43.3%)	
At least two drinks per day	49/399 (12.3%)	27/242 (11.1%)	22/157 (14%)	
Tobacco				0.778
Never	206/419 (49.2%)	132/261 (50.6%)	74/158 (46.8%)	
Current	8/419 (1.9%)	5/261 (1.9%)	3/158 (1.9%)	
Former	205/419 (48.9%)	124/261 (47.5%)	81/158 (51.3%)	
Body Mass Index, kg/m^2^				< 0.001
< 30	248/436 (56.9%)	139/275 (50.5%)	109/161 (67.7%)	
30- < 40	157/436 (36%)	110/275 (40%)	47/161 (29.2%)	
≥ 40	31/436 (7.1%)	26/275 (9.5%)	5/161 (3.1%)	
Hypertension	218 (49.5%)	129 (46.4%)	89 (54.9%)	0.084
Chronic Obstructive Pulmonary Disease	15 (3.4%)	10 (3.6%)	5 (3.1%)	0.776
Asthma	26 (5.9%)	17 (6.1%)	9 (5.6%)	0.810
Diabetes mellitus	116 (26.4%)	68 (24.5%)	48 (29.6%)	0.235
Mild to severe chronic renal failure ≥ 265 µmol/L	7/438 (1.6%)	5/277 (1.8%)	2/161 (1.2%)	0.999
Congestive heart failure	13 (3%)	8 (2.9%)	5 (3.1%)	0.999
Solid tumor with or without metastases within 5 years	24 (5.5%)	14 (5%)	10 (6.2%)	0.613
Hematological malignancy	14 (3.2%)	7 (2.5%)	7 (4.3%)	0.299
Charlson comorbidity index ≥ 1	193/438 (44.1%)	108 (38.8%)	85/160 (53.1%)	0.004
Simplified Acute Physiology Score II	34.5(28, 43) [438]	32 (26, 42) [276]	36.5 (30, 45.7) [162]	< 0.001
Sequential Organ Failure Assessment score	4 (2.75, 6)	4 (3, 5.75)	4 (2.2, 7)	0.068
C-reactive Protein maximum blood level, mg/L	152.2 (99,218.2) [306]	148 (94.2, 213.5) [201]	162.9 (109, 225) [105]	0.111
Most invasive respiratory support				< 0.001
IMV	261 (59.3%)	135 (48.6%)	126 (77.8%)	
NIV	38 (8.6%)	28 (10%)	10 (6.2%)	
HFOT	141 (32.1%)	115 (41.4%)	26 (16%)	
Most severe P/F ratio				0.079
[200; 300[	17/433 (3.9%)	10/272 (3.7%)	7/161 (4.3%)	
[100; 200[	178/433 (41.1%)	123/272 (45.2%)	55/161 (34.2%)	
< 100	238/433 (55%)	139/272 (51.1%)	99/161 (61.3%)	
Patients receiving IMV				
Duration, days	17 (9, 26) [251]	13.3 (8,19) [133]	21 (13.3, 33) [118]	< 0.001
Minimum of Static Compliance of the Respiratory System, ml per cm of water	26.5 (16.3, 33.7) [150]	26 (14, 35) [77]	27 (20, 33) [73]	0.891
PEEP max, cm of water	14 (12, 15) [244]	14 (11.7, 15) [124]	14 (12, 16) [120]	0.460
Maximum of Plateau Pressure, cm of water	29 (25, 32) [216]	28 (25, 32) [109]	30 (26, 33) [107]	0.064
Prone Positioning	175/261 (67%)	86/135 (63.7%)	89/126 (70.6%)	0.234
Neuromuscular blockade	231/261 (88.5%)	115/135 (85.2%)	116/126 (92.1%)	0.082
Inhaled nitric oxide	36/259 (13.9%)	12/133 (9%)	24/126 (19%)	0.020
VV ECMO	15/260 (5.8%)	7/134 (5.2%)	8/126 (6.3%)	0.697
VV ECMO duration, mean (SD), days	11.3 (7.6) [15]	11.6 (9.6) [7]	11.1 (6.2) [8]	0.999
IMV weaning				
Tracheal re-intubation after weaning failure	42/249 (16.9%)	19/130 (14.6%)	23/119 (19.3%)	0.321
Tracheotomy	47/261 (18%)	13/135 (9.6%)	34/126 (27%)	< 0.001
Tracheotomy duration, days	16.5 (10, 31.2) [44]	14 (10, 16) [12]	19 (11.5, 35) [32]	0.097
NIV as most invasive respiratory support				
PEEP max, cm of water	8 (6.5, 10) [31]	8 (6,10) [23]	9 (8, 10) [8]	0.200
FiO_2_ max, %	70 (60, 84) [36]	70 (60, 88) [26]	75 (60, 81.5) [10]	0.693
NIV duration, days	4.5 (2.5, 6.7) [34]	4 (2, 6) [26]	5.5(4, 7) [88]	0.335
HFOT as most invasive respiratory support				
Flow max, L/min	50 (50, 60) [131]	50 (50, 60) [109]	50 (50, 60) [22]	0.553
FiO_2_ max, %	70 (60, 88.7) [134]	70 (60, 88.3) [110]	70 (60, 86.3) [24]	0.384
HFOT duration, days	6 (4, 8) [135]	6 (4, 8) [110]	7 (5, 11) [25]	0.028
Other organ support				
Vasopressors	199 (45.2%)	97 (34.9%)	102 (63%)	< 0.001
Vasopressor duration, days	5 (3,11) [195]	4 (2,7) [94]	8 (3,15) [101]	< 0.001
Inotropic agent	14 (3.2%)	5 (1.8%)	9 (5.6%)	0.030
Renal replacement therapy	25/439 (5.7%)	10 (3.6%)	15/161 (9.3%)	0.013
Specific treatments for COVID-19				
Antiviral therapies	94 (21.4%)	51 (18.3%)	43 (26.5%)	0.043
Immunomodulatory therapies	25 (5.7%)	10 (3.6%)	15 (9.3%)	0.013
Corticosteroids	260 (59.1%)	163 (58.6%)	97 (59.9%)	0.798
Time from ICU admission to hospital discharge, days	23 (14, 45)	19 (13, 31)	41 (20, 60.7)	< 0.001
Length of ICU stay, days	12 (7, 28)	9.5 (6, 18)	24 (12, 40.7)	< 0.001

Data are expressed as n (%), [N0: Number of patients with available data in case of missing data], n/N0, or median (IQR), unless stated otherwise

FiO_2_ max, maximum of fraction of inspired oxygen; HFOT, high flow oxygen therapy; ICU, intensive care unit; IMV, invasive mechanical ventilation; NIV, non-invasive mechanical ventilation; P/F ratio, partial pressure of arterial oxygen divided by fraction of inspired oxygen; PEEP max, maximum of positive end-expiratory pressure; VV ECMO, veno-venous extracorporeal membrane oxygenation

**Table 2:** Description of baseline CT according to the presence or absence of fibrotic changes

	All patients, N = 440	No fibrotic changes, N = 278	Fibrotic changes, N = 162	*P*-value
Time between ICU admission and Baseline CT, days	0 (-2,0)	0 (-2,0)	0 (-1,0)	0.004
Timing of Baseline CT, in intervals (0 = ICU admission), days				0.093
[-15;-5[	18 (4.1%)	11 (4%)	7 (4.3%)	
[-5;5[	374 (85%)	243 (87.3%)	131 (80.9%)	
[5;10[	21 (4.8%)	13 (4.7%)	8 (4.9%)	
[10;21]	27 (6.1%)	11 (4%)	16 (9.9%)	
Number of early fibrotic signs				< 0.001
0	284 (64.5%)	197 (70.9%)	87 (53.7%)	
1	79 (18.8%)	48 (17.3%)	31 (19.1%)	
2	49 (11.1%)	24 (8.6%)	25 (15.4%)	
≥ 3	28 (6.4%)	9 (3.2%)	19 (11.7%)	
Type of lesion				
Traction bronchiectasis (fibrotic sign)	80 (18.2%)	30 (10.8%)	50 (30.9%)	< 0.001
Loss of lung volume (fibrotic sign)	62 (14.1%)	29 (10.4%)	33 (20.4%)	0.004
Lung architectural distortion (fibrotic sign)	118 (26.8%)	64 (23%)	54 (33.3%)	0.019
Honeycombing (fibrotic sign)	2 (0.5%)	0 (0%)	2 (1.2%)	0.135
Ground-glass opacity	438 (99.5%)	277 (99.6%)	161 (99.44%)	0.999
Consolidation	378 (85.9%)	237 (85.3%)	141 (87%)	0.604
Crazy Paving	115 (26.1%)	61 (21.9%)	54 (33.3%)	0.009
Bronchial distortion within COVID-19 opacities	183 (41.6%)	93 (33.5%)	90 (55.6%)	< 0.001
Traction bronchiectasis outside COVID-19 opacities	21 (4.8%)	4 (1.4%)	17 (10.5%)	< 0.001
Reticulation	269 (61.1%)	157 (56.5%)	112 (69.1%)	0.009
Parenchymal band	219 (49.8%)	143 (51.4%)	76 (46.9%)	0.360
Translobular line	242 (55%)	157 (56.5%)	85 (52.2%)	0.415
Posterior irregular or thickened pleural interface	280 (63.6%)	170 (61.2%)	110 (67.9%)	0.156
Pleural effusion	155 (35.2%)	97 (34.9%)	58 (35.8%)	0.847
Reversed halo	18 (4.1%)	10 (3.6%)	8 (4.9%)	0.493
Halo	10 (2.3%)	5 (1.8%)	5 (3.1%)	0.509
Indicators of bacterial superinfection	49 (11.1%)	32 (11.5%)	17 (10.5%)	0.744
Tree-in-bud nodules	9 (2%)	4 (1.4%)	5 (3.1%)	0.299
Lobar or segmental single consolidation	27 (6.1%)	19 (6.8%)	8 (4.9%)	0.424
Endobronchial secretion	22 (5%)	15 (5.4%)	7 (4.3%)	0.618
Iodinated contrast agent injection				0.515
No	160 (36.4%)	96 (34.5%)	64 (39.5%)	
Yes	260 (59.1%)	168 (60.4%)	92 (56.8%)	
Both pre-contrast and post-contrast images	20 (4.5%)	14 (5%)	6 (3.7%)	
Pulmonary embolism	42/247 (17%)	25/164 (15.2%)	17/83 (20.5%)	0.301
Endotracheal tube	63 (14.3%)	27 (9.7%)	36 (22.2%)	< 0.001
Evidence of Barotrauma	14/438 (3.2%)	6/277 (2.2%)	8/161 (5.2%)	0.108
Lung involvement				
Visual evaluation, %	44.7 (28.1, 60.6)	37.5 (23.7, 52.9)	51.8 (38.9, 69.8)	< 0.001
Visual lung involvement, %, in categories				< 0.001
< 10%	18 (4.1%)	15 (5.4%)	3 (1.9%)	
10–25%	72 (16.4%)	59 (21.2%)	13 (8%)	
25–50%	172 (39.1%)	119 (42.8%)	53 (32.7%)	
50–75%	128 (29.1%)	68 (24.5%)	60 (37%)	
> 75%	50 (11.4%)	17 (6.1%)	33 (20.4%)	
CT pneumonia analysis % of opacities, %	49.5 (33.8, 64.4) [432]	44.3 (28.8, 61.1) [273]	57.3 (41.7, 69.8) [159]	< 0.001
Opacity score (0–20)	12 (9,15) [432]	11 (9,14) [273]	14 (11,17) [159]	< 0.001

Data are expressed as n (%), [N0: Number of patients with available data in case of missing data], n/N0, or median (IQR), unless stated otherwise. CT: Chest computed tomography; ICU: Intensive Care Unit

### Primary outcome

**Fig. 2:**
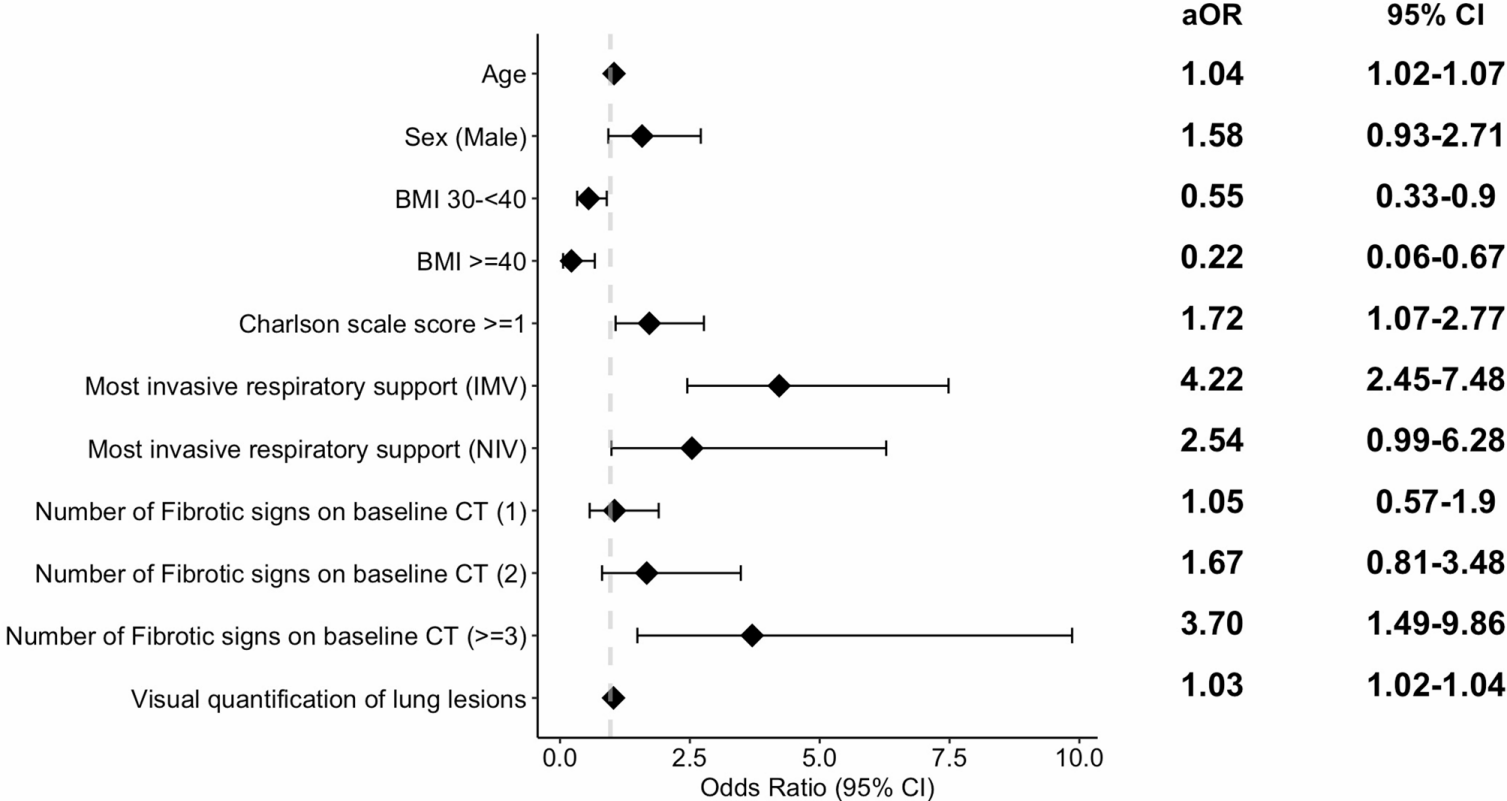
Primary analysis: multivariate analysis for predictive factors of fibrotic changes 6 months after ICU discharge. Each additional year of age and each additional percent of lung involvement on baseline CT was associated with an increase of 4% and 3% of developing fibrotic changes at 6 months, respectively. aOR; adjusted odds ratio, BMI, Body mass Index; CT, Computed tomography; IMV, Invasive Mechanical Ventilation; NIV, Non-invasive ventilation

By multivariate analysis, the odds of FC at follow-up were significantly higher in older patients, those with a body mass index (BMI) below 30 kg/m^2^, a Charlson comorbidity score of one or higher at ICU admission, those who required invasive mechanical ventilation (IMV) during the ICU stay, and those who exhibited at least 2 early fibrotic signs and greater lung involvement at baseline CT (Fig. 2) and additional file (Table S3).

**Fig. 3:**
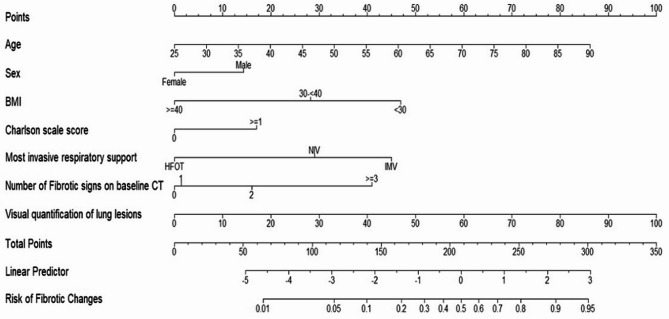
Nomogram for predicting fibrotic changes 6 month after ICU discharge. For example, a 60-year-old [46 points] male patient [14 points] with a BMI < 30 [47 points], a Charlson comorbidity score of 1 [17 points], requiring invasive mechanical ventilation in the ICU [45 points], and a baseline CT showing two signs of fibrosis [16 points] and lung involvement of 55% [55 points], has an estimated risk of developing fibrotic changes within 6 months of 74% [240 points]. BMI, Body Mass index; CT, Computed tomography

The nomogram incorporating these factors (Fig. 3) yielded a robust prediction of FC at six months post-ICU discharge, with an AUC of 80.6% (95% CI: 76.4–84.8) for the entire cohort. Specifically, the AUC was 81.2 and 79.8 for the training and the test set respectively (additional file, Fig. S3 and Table S4).

### Secondary outcomes

**Fig. 4:**
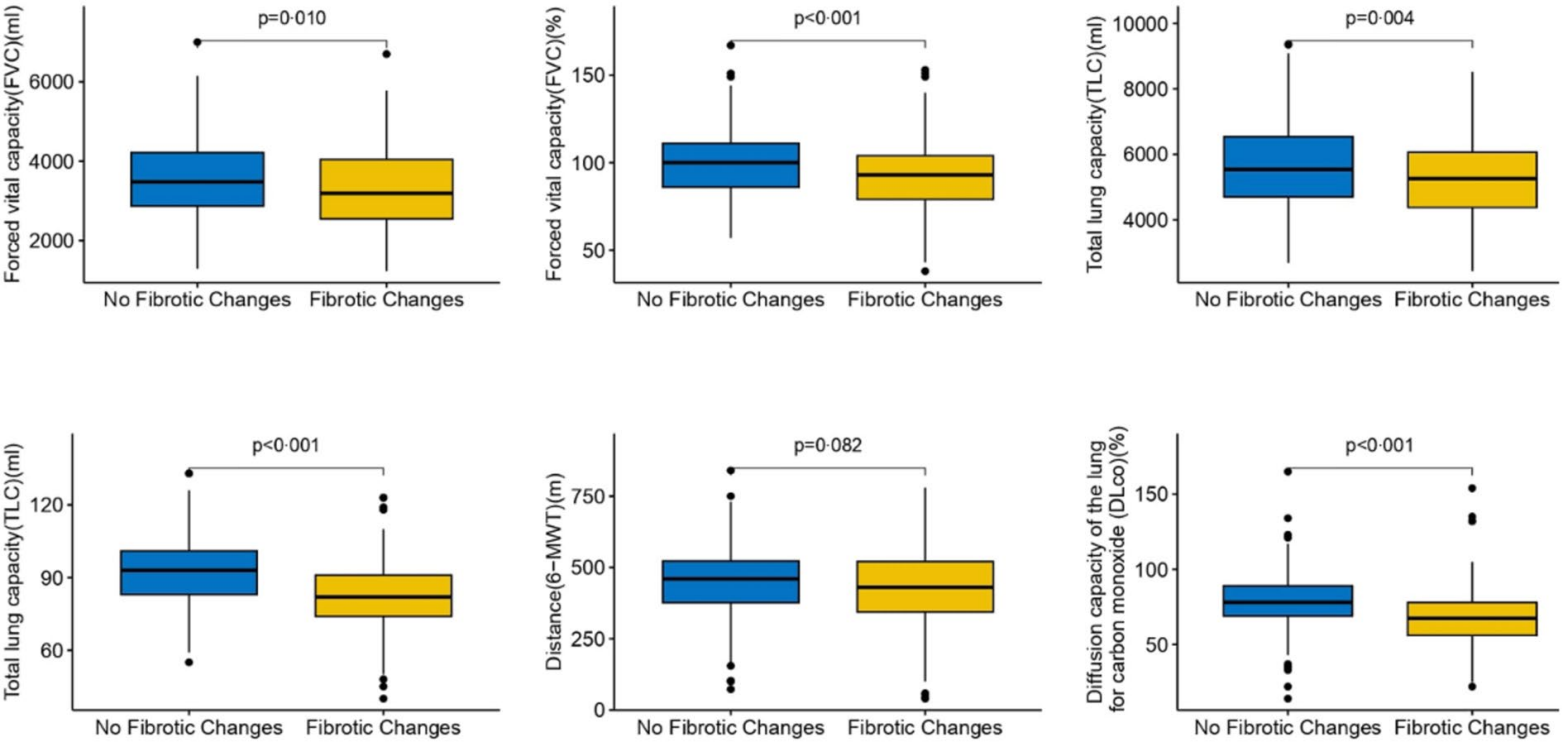
Main respiratory outcomes according to lung fibrotic changes 6 months after ICU discharge

Regarding follow-up CT evaluation (Table 3), the median interval from baseline CT was 6.5 months (6.1–7.2), the most frequent radiological pattern was late OP with or without distortion, observed in 206 patients (47.2%). In the FC group, visual lung involvement was higher (13.7% [8.1–24.1] vs. 2.5% [1.1–6.3]; p < 0.001). The visual quantification of lung lesions decreased with increasing time elapsed since ICU discharge (p = 0.004), whereas the prevalence of FC did not vary significantly (additional file, Figs. S4 and S5, respectively). Patients in the FC group at 6 months post-ICU discharge more frequently presented shortness of breath according to the mMRC and had significantly lower SpO_2_ at rest and after exertion (additional file, Tables S5 and S6). They also had significantly more impaired forced vital capacity (FVC), total lung capacity (TLC), diffusion capacity of the lung for carbon monoxide (DL_CO_), and carbon monoxide transfer coefficient (K_CO_) (Fig. 4). Regarding QoL, only the physical domain of the SF-36 was significantly more impaired for patients with FC (additional file, Fig. S6), with no differences observed in the physical or mental summary scores (additional file, Fig. S7) or in the VSRQ (additional file, Table S5).

**Table 3:** Description of follow-up CT according to fibrotic changes after ICU discharge

	All patients, N = 440	No Fibrotic Changes, N = 278	Fibrotic Changes, N = 162	*P*-value
Time between ICU discharge and follow-up CT, months	6.5 (6.1, 7.2)	6.4 (5.9, 6.9)	6.7 (6.2, 7.5)	**0.003**
Intervals between ICU discharge and follow-up CT, months				**0.017**
[2;4[	10 (2.3%)	10 (3.6%)	0 (0%)	
[4;6[	93 (21.1%)	63 (22.7%)	30 (18.5%)	
[6;8[	289 (65.7%)	180 (64.7%)	109 (67.3%)	
≥ 8	48 (10.9%)	25 (9%)	23 (14.2%)	
Type of lesion				
Traction bronchiectasis	149 (33.9%)	0 (0%)	149 (92%)	** < 0.001**
Loss of lung volume	79 (18)	0 (0%)	79 (48.8%)	** < 0.001**
Lung architectural distortion	107 (24.3%)	0 (0%)	107 (66%)	** < 0.001**
Honeycombing	10 (2.3%)	0 (0%)	10 (6.2%)	** < 0.001**
Number of fibrotic signs				** < 0.001**
0	278 (63%)	278 (100%)	0 (0%)	
1	58 (13%)	0 (0%)	58 (36%)	
2	35 (8%)	0 (0%)	35 (22%)	
≥ 3	69 (16%)	0 (0%)	69 (43%)	
Combined traction bronchiectasis and architectural distortion	95 (22%)	0 (0%)	95 (58.6%)	** < 0,001**
Predominant lesional pattern				** < 0.001**
Anterior Fibrosis	30/437 (6.9%)	0/275 (0%)	30 (18.5%)	
Other Fibrosis	20/437 (4.9%)	0/275 (0%)	20 (12.3%)	
Minimal Lesions	26/437 (5.9%)	26/275 (9.5%)	0 (0%)	
Late Organizing Pneumonia with distortion	103/437 (23.6%)	11/275 (4%)	92 (56.8%)	
Late Organizing Pneumonia without distortion	103/437 (23.6%)	90/275 (32.7%)	13 (8%)	
Complete resolution	53/437 (12.1%)	53/275 (19.3%)	0 (0%)	
Ground-glass	102/437 (23.3%)	95/275 (34.5%)	7 (4.3%)	
Type of ground-glass opacities				0.489
Mosaic attenuation	14/102 (13.7%)	12/95 (12.6%)	2/7 (28.6%)	
Melting sugar appearance	83/102 (81.4%)	78/95 (82.1%)	5/7 (71.4%)	
Non specific	5/102 (4.9%)	5/95 (5.3%)	0/7 (0%)	
Lung involvement				
Visual Evaluation, %	5.6 (1.8, 13.7) [428]	2.5 (1.1, 6.3) [268]	13.7 (8.1, 24.1) [160]	** < 0.001**
Percentage visual lung involvement, in categories				** < 0.001**
[0–10[	278/428 (65.5%)	225/268 (84%)	53/160 (33.1%)	
[10–25[	100/428 (23.4%)	33/268 (12.3%)	67/160 (41.9%)	
[25; 50[	36/428 (8.4%)	9/268 (3.4%)	27/160 (16.9%)	
[50–75[	13/428 (3.3%)	1/268 (0.4%)	12/160 (7.5%)	
[75–100]	1/428 (0.2%)	0/268 (0%)	1/160 (0.6%)	
Presence of Emphysema	131/434 (30.2%)	72/274(26.3)	59/160 (36.9%)	**0.020**
Median extent of Emphysema (% of Low Attenuation Area over total lung volume)	1.0 (0.3, 2.8) [118]	1.4 (0.3,2.9) [65]	0.7 (0.3, 2.5) [53]	0.406

Data are expressed as n (%), [N0: Number of patients with available data in case of missing data], n/N0, or median (IQR), unless stated otherwise. CT: Chest computed tomography; ICU: Intensive Care Unit

## Discussion

To our knowledge, this study presents the largest cohort of COVID-19 ARDS survivors undergoing chest CT evaluation at a median interval of 6.5 months post-ICU discharge. In our population, FC were observed in 36.8% of the 440 survivors, a prevalence that is hard to compare with existing literature due to variations in the definitions of fibrosis and the timing of follow-up CT [4, 19, 20]. Despite a restrictive definition, our cohort exhibited a high frequency of FC, somewhat comparable to the rate of 39% observed at 4 months in the ARDS subgroup of the COMEBAC cohort [21]. The elementary lesions used, conforming to Fleischner Society’s glossary from 2008 (which was in use at the time of the image interpretation) and consistent with the 2024 updated version [22], are theoretically non-regressive when identified in a chronic setting [12]. This may account for the stable prevalence of FC observed between the earliest and latest follow-up CTs in our study, despite a reduction in the overall burden of lesions. Additionally, studies have confirmed that FC identified at 6 months remained up to 24 months post-ARDS-COVID [21, 22].

Anterior fibrosis emerged as the predominant pattern in 30 (19%) of the 160 evaluable patients with FC. IMV carries a risk of inducing or perpetuating lung injury, corresponding to the term "Ventilator-Induced Lung Injury (VILI)," whose effects may be limited by applying protective ventilation [25]. During ARDS, the anterior zones of the lungs are most accessible to IMV due to consolidation of posterior dependent lung regions. This increases the risk of regional lung overdistension in the nondependent anterior areas, potentially leading to lung injuries. This anterior localization of fibrotic damage could thus correspond to the sequelae of VILI among COVID-19-ARDS survivors who likely suffered the most in terms of ventilation, akin to survivors of ARDS of other origins who underwent prolonged IMV [26]. In our study, the duration of IMV and the proportion of tracheotomy for IMV weaning were significantly higher in the FC group. The measurement of airway plateau pressure, used to evaluate end-inspiratory intra-alveolar pressure, was higher in the FC group, albeit not statistically significant (p = 0.064), with a median of 30 cmH2O (IQR 26–33), a threshold beyond which the risk of VILI is significant [25]. Prone positioning enhances the aeration of the dependent regions, promoting uniform ventilation and reducing the risk of overdistension [25], however we observed no protective effect against FC in our cohort.

Late OP with or without distortion was the most commonly encountered CT pattern, possibly corresponding to the specific residual impact of COVID-19 as demonstrated in histologic specimen from COVID-19 survivors demonstrating radiological sequelae [25, 26]. Indeed, GGO and consolidations tend to regress by one year [19].

The association between OP and FC could stem from the severity of alveolar damage inflicted by the viral infection, influenced by the patient's inflammatory or immune response, and may be further compounded by impaired or delayed repair mechanisms, which are exacerbated by specific risk factors [3]. We suppose that the association of FC with a Charlson comorbidity score may be attributed to the increased likelihood that more frail patients will require IMV and experience delayed or incomplete repair. Some of the risk factors for FC identified here have also been identified in other cohorts with non-severe or mixed severity COVID-19. Increasing age appears to significantly elevate the risk of FC, with a threshold identified at 50 years in one study [4]. The increased risk related to the type of ventilatory support initially used (non-invasive ventilation or IMV) has also been reported [20, 27]. Interestingly, 26 of 162 patients with FC (16%) had HFOT as maximal respiratory support. The higher prevalence of emphysema observed in the FC group—despite comparable rates of tobacco use and somewhat similar extent—may be explained by the greater use of IMV, as well as more severe acute disease in this group, as previously suggested in the literature [20]. In this study, the baseline CT played a crucial role in identifying patients at risk of FC. The association between the extent of initial lesions and FC has already been demonstrated, whether through a semi-quantitative analysis by the severity score [4, 29] or automated quantification [30]. However, the relevance of identifying early signs of potential fibrosis had not yet been reported, with an adjusted OR of 3.7 (95%CI 1.5–10) for predicting FC. We noted that early fibrosis was absent in over half of the patients who later developed FC. Conversely, these signs regressed in under a third of the patients without FC at follow-up, underscoring both the potential for repair and the risk of developing FC later during the ICU stay.

Interestingly, obesity (BMI > 30 kg/m^2^) appeared to protect against FC, despite its association with an increased risk of COVID-19 ARDS [31]. The protective effect of obesity on non-COVID-19 ARDS mortality, previously termed “the obesity paradox” [32], remains uncertain in ARDS-COVID-19, since one meta-analysis found no significant impact on the survival of obese patients [31]. In obese patients ventilated invasively, hypoxemia is often the consequence of atelectasis and mismatch between ventilation and perfusion [33]. This may lead to an overestimation of ARDS severity related to collapsed but unharmed lung areas that exhibit no scarring once the atelectasis is resolved. Furthermore, inflammatory and metabolic factors linked to obesity could account for lesser lung involvement and ultimately fewer sequelae [30, 31].

Our study suggests a significant clinical impact of FC, since patients with FC had increased dyspnea, lower post-exertion SpO_2_, and covered 29 m less on the 6MWT, although this difference borders statistical significance, it remains clinically relevant, particularly given that the observed effect was not explained by the higher prevalence of emphysema in the FC group [34]. These clinical characteristics could be linked to FC and possible exertional hypoxemia. However, it is likely that the consequences of intensive care on the functional state, notably neuromuscular, had a significant impact, since patients in the FC group more frequently required IMV, and had a hospital stay more than twice as long as patients without FC. Nevertheless, direct impairment from FC should not be ruled out, as PFT revealed a restrictive respiratory profile, along with lower DLco and no elevation of Kco, further advocating for a functional reality linked with the FC on follow-up CT [35]. In our study, the residual lesions in the FC group affected a median of 14% (8–24) of the lungs, surpassing the 10% threshold proposed by Mehta et al. to deem lesions symptomatically significant [1]. The impact on QoL across the cohort was more significant in physical than in mental terms, comparable to what is usually described in the post-ARDS recovery period [36]. However, the QoL related to the physical state (physical role) was more markedly diminished in the FC group.

The potential impact of FC on clinical outcomes highlights the possible benefit of antifibrotic treatments and the need for early identification of high-risk patients [37]. A nomogram derived from our cohort can evaluate the risk of FC, enabling tailored assessment of patients based on initial data, including CT. Visual lesion extent on baseline CT in patients with FC varied with the time from admission to ICU, indicating that scans performed more than five days before ICU admission may underestimate lesion burden. Maximal lesion quantification occurred between five and ten days, suggesting this as the most suitable interval for future assessment, at a time where all the selected risk factors are readily accessible.

Our study has several limitations. Firstly, we cannot rule out that FC on CT preceded ARDS and COVID-19. However, the exclusion of patients with advanced respiratory histories and seemingly pre-existing interstitial abnormalities limited this possibility. Secondly, our evaluation at 6 months after ICU discharge may overestimate FC, as some residual non-fibrotic lesions may still improve over time. Current data suggest that FC would remain stable. Thirdly, it is difficult to distinguish between symptoms arising from FC and those related to post intensive care syndrome in these patients. A further evaluation more distant from ICU discharge could yield a better understanding of this issue. Finally, the model for predicting FC may not be generalizable to ARDS of other aetiologies due to the specificities of COVID-19 ARDS even if these ones are still debate [38] 39. However, the identified risk factors should be taken into consideration in future studies.

## Conclusion

Post COVID-19 ARDS FC were frequently observed at 6 months post-ICU discharge. Two major lesion patterns were identified, namely anterior fibrosis, suggesting sequelae from IMV, and more commonly, a pattern of late OP with varying degrees of distortion, which may correspond to viral sequelae. Furthermore, our study highlighted clinical and radiological factors which could enable the early identification of patients who may benefit from targeted follow-up and specific management.

## Supplementary Information

Additional file1 (DOCX 1084 kb)

## Data Availability

Study data will be made available upon reasonable requests made to the corresponding author.

## Abbreviations

6MWT: 6-Minute walk test
ARDS: Acute respiratory distress syndrome
AUC: Area under the curve
BMI: Body mass index
CIs: Confidence intervals
COVID-19: Corona Virus Disease-2019
CT: Computed tomography
DLco: Diffusion capacity of the lung for carbon monoxide
FC: Fibrotic changes
FiO_2_: Fraction of inspired Oxygen
FVC: Forced vital capacity
GGO: Ground glass opacities
HFOT: High flow nasal oxygen
ICU: Intensive care unit
ILD: Interstitial lung disease
IMV: Invasive mechanical ventilation
IPF: Idiopathic pulmonary fibrosis
IQR: Interquartile ranges
Kco: Carbon monoxide transfer coefficient
mMRC: Medical Research Council dyspnea scale
OP: Organizing pneumoniae
ORs: Odds ratios
PFT: Pulmonary function tests
QoL: Quality of life
SAPS II: Simplified Acute Physiology Score II
SD: Standard deviation
SF-36: Short Form Health Survey modified 36
TLC: Total lung capacity
VILI: Ventilator-induced lung injury
VSRQ: Visual Simplified Respiratory Questionnaire
