# Supplementary material for: ICU predictive factors of fibrotic changes following COVID-19 related ARDS: a RECOVIDS substudy
Source: Ann Intensive Care. 2025 Nov 4;15:177. doi: 10.1186/s13613-025-01577-2 (PMC12583252; doi:10.1186/s13613-025-01577-2)
Supplement: Supplementary file 1 — Additional file1 (DOCX 1084 kb) [file 13613_2025_1577_MOESM1_ESM.docx]

**ICU predictive factors of fibrotic changes following COVID-19 related ARDS: a RECOVIDS substudy**

**Supplemental Online Content**

# eMethods 1. RECOVIDS main study non-inclusion criteria p. 2

# eMethods 2. Main secondary radiological signs outcomes p. 3

# eMethods 3. Methods of calculation of lung lesions extension p. 4

**eTable 1.** Primary analysis: results of the bivariate analysis for predictive factors of **p. 5** fibrotic changes 6 months after ICU discharge

**eFigure 1.** Comparison of ROC curves for evaluation of the best method to assess baseline **p. 6** lung involvement to predict pulmonary fibrotic changes at follow-up: visual quantification vs automated lung opacity percentage

**eTable 2** Details on lung involvement on baseline CT according to the type **p. 7-8** of lung zone studied and the method of quantification

**eFigure 2.** Visual quantification of lung lesions identified at baseline in the fibrotic **p.9** changes group according to time interval between CT acquisition and ICU admission

**eTable 3.** Primary analysis: results of the multivariate analysis (full model) for predictive **p. 10** factors of fibrotic changes 6 months after ICU discharge

**eFigure 3.** ROC Curves for evaluating the final model accuracy in predicting fibrotic **p. 11** changes 6 months after ICU Discharge, calibration plot and ROC Curve (AUC) for the final logistic regression model

**eTable 4.** Descriptive comparison of final model variables between training and test sets **p.12**

**eFigure 4.** Global visual quantification of lung lesions identified on follow-up CT **p.13** in the entire cohort according to time interval between ICU discharge and follow-up CT

**eFigure 5.** Prevalence of fibrotic changes on follow-up according to time interval between **p.14** ICU discharge and follow-up CTeTable 5. Respiratory outcomes 6 months after ICU discharge according to fibrotic changes **p. 15**

# eTable 6. Multivariable median quantile regression analysis: effect of fibrotic changes p.16 and emphysema during follow-up on 6MWT walking

# eFigure 7. Quality of life according to the 8 subscales of the SF-36 questionnaire and fibrotic p. 17 changes status 6 months after ICU discharge

# eFigure 8. SF-36 General health assessment, physical and mental summary scores at 6 p. 18 months after ICU discharge according to fibrotic changes

# Supplementary Table 1. Sensitivity analysis comparing study population with the p. 19 subjects excluded due to lack of follow-up Chest CT

# Supplementary Table 2. Prevalence of traction bronchiectasis and architectural p. 20 distortion combinedeMethods 1. RECOVIDS main study non-inclusion criteria

| Age <18 years |
| --- |
| Limited autonomy prior to hospital admission defined by a walking perimeter <50m or WHO performance status 3 or 4 |
| A history of chronic respiratory insufficiency defined by the use of long-term oxygen therapy or non-invasive home ventilation (except for patients with sleep apnoea and/or obesity hypoventilation syndrome) |
| A history of central or peripheral neurological disorders limiting motor autonomy and impairing the ability to perform the walk test or pulmonary function tests |
| Refusal to participate |
| Patients not affiliated or not benefitting from national health insurance, in accordance with French legislation |
| Patients under legal protection: guardianship, curatorship or protected adults |
| Inability to comprehend and consent to the study |

# eMethods 2. Main secondary radiological signs outcomes

# Ground glass opacities (GGO)

# Consolidation

# Crazy paving

# Reticulation

# Parenchymal bands

# Translobular lines

# Irregularities or thickening of the posterior pleural line

# Pleural fluid

# Halo sign and reverse halo sign

# Indicators of bacterial superinfection (only baseline CT)

# Pulmonary embolism (only baseline CT)

# Barotrauma (only baseline CT)

**eMethods 3. Methods of calculation of lung lesions extension**

A-Visual calculation:

The extent of lesions was quantified in increments of 5% ranging from 0 to 100%, for the right and left lungs, each divided into 4 areas, using anatomical landmarks including the aortic arch, the carina, and the ostium of the right inferior pulmonary vein.

Average visual extent was subsequently calculated

B-Automated calculation:

Siemens Healthineers' technology independently processed the CTs using the CT Pneumonia Analysis application on Syngo.Via (Siemens Healthineers, Erlangen, Germany).

Opacity score was calculated for each lobe (0: no opacity, 1:≤25%, 2:>25-≤50, 3:>50-≤75, 4:>75%) (total score 0-20), and extension and volume of opacity and high opacity for the whole lung, and each lobe was recorded only for baseline CTs achieving a quality score of 1, 2, or 3.

# eTable 1. Primary analysis: results of the bivariate analysis for predictive factors of fibrotic changes 6 months after ICU discharge

|  | **OR Bivariate (95%CI)** | **P-value** |
| --- | --- | --- |
| **Age** | 1.05 (1.03-1.07) | **<0.001** |
| **Sex (Male)** | 1.81 (1.17-2.84) | **<0.008** |
| **BMI** | .. | .. |
| **<30** | **ref** | .. |
| **30-<40** | 0.54 (0.35-0.83) | **<0.004** |
| **≥40** | 0.25 (0.08-0.61) | **<0.005** |
| **Charlson score** | .. | .. |
| **0** | .. | .. |
| **≥ 1** | 1.78 (1.21-2.65) | **<0.004** |
| **Most severe P/F ratio** | .. | .. |
| **[200; 300[** | **ref** | .. |
| **[100; 200[** | 0.64 (0.23-1.84) | 0.387 |
| **<100** | 1.02 (0.37-2.89) | 0.973 |
| **Most invasive respiratory support** | .. | .. |
| **HFOT** | **ref** | .. |
| **IMV** | 4.13 (2.56-6.85) | **<0.001** |
| **NIV** | 1.58 (0.66-3.58) | 0.285 |
| **SAPSII** | 1.02 (1.01-1.04) | **<0.002** |
| **SOFA** | 1.07 (0.99-1.14) | **0.056** |
| **ICU acquired pneumonia (yes)** | 3.35 (2.21-5.12) | **<0.001** |
| **Antiviral therapies (yes)** | 1.61 (1.01-2.55) | **0.044** |
| **Corticosteroids (yes)** | 1.05 (0.71-1.57) | 0.798 |
| **Other Immunomodulatory therapies (yes)** | 1.17 (0.78-1.76) | 0.433 |
| **Nosocomial Pneumonia (yes)** | 2.67 (1.22-5.98) | **0.014** |
| **Baseline chest CT data** | .. | .. |
| **Consolidation (yes)** | 1.16 (0.66-2.08) | 0.604 |
| **Crazy Paving (yes)** | 1.78 (1.15-2.74) | **<0.009** |
| **Bronchial distortion within COVID-19 opacities** | 2.49 (1.67-3.71) | **<0.001** |
| **Visual evaluation of lung involvement** | 1.03 (1.02-1.04) | **<0.001** |
| **Number of early fibrotic signs** | .. | .. |
| **0** | **ref** | .. |
| **1** | 1.46 (0.86-2.45) | 0.149 |
| **2** | 2.36 (1.27-4.38) | **<0.006** |
| **≥3** | 4.78 (2.13-11.5) | **<0.001** |

CI, confidence interval; CT, computed tomography; HFOT, high flow oxygen therapy; ICU, intensive care unit; IMV, invasive mechanical ventilation; NIV, non-invasive mechanical ventilation; P/F ratio, partial pressure of arterial oxygen divided by fraction of inspired oxygen; SPASII, Simplified Acute Physiology Score II; SOFA, Sequential Organ Failure Assessment score.

# eFigure 1. Comparison of ROC curves for evaluation of the best method to assess baseline lung involvement to predict pulmonary fibrotic changes at follow-up: visual quantification vs automated lung opacity percentage


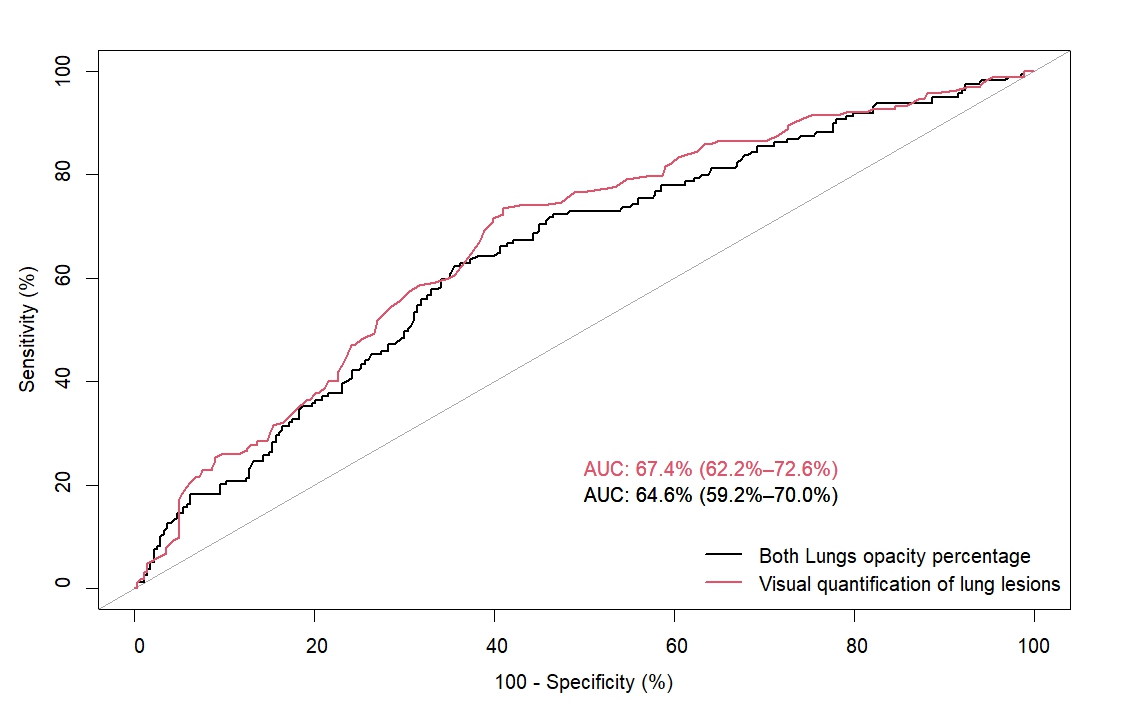


# eTable 2. Details on lung involvement on baseline CT according to the type of lung zone studied and the method of quantification

|  | **All patients, N=440** | **No Fibrotic Changes, N=278** | **Fibrotic Changes, N=162** | **P-value** |
| --- | --- | --- | --- | --- |
| **Area involvement, visual quantification, %** | .. | .. | .. | .. |
| **Area 1** | 30 (10, 60) | 20 (10, 50) | 40 (15, 70) | **<0.001** |
| **Area 2** | 50 (25, 75) | 40 (20, 70) | 60 (40, 85) | **<0.001** |
| **Area 3** | 50 (30, 70) | 40 (26, 60) | 65 (40, 78.7) | **<0.001** |
| **Area 4,** | 50 (30, 66.3) | 40 (25, 60) | 57.5 (30, 75) | **<0.001** |
| **Area 5** | 20 (5, 40) | 15 (5, 30) | 30 (10, 60) | **<0.001** |
| **Area 6** | 40 (20, 70) | 35 (20, 65) | 57.5 (30, 75) | **<0.001** |
| **Area 7** | 55 (30, 75) | 50 (30, 70) | 70 (45, 83.7) | **<0.001** |
| **Area 8** | 45 (25, 70) | 40 (20, 65) | 50 (35, 80) | **<0.001** |
| **Both lungs, involvement** | .. | .. | .. | .. |
| **Both Lungs, opacity volume, ml** | 1599.9 (1083.1, 2241.9) [432] | 1430.1 (924, 2059.4) [273] | 1962.1 (1445.3, 2564.9) [159] | **<0.001** |
| **Both Lungs, High opacity volume, ml** | 347.5 (182.5, 591.7) [432] | 302 (162.6, 514.2) [272] | 447.6 (250.8, 654.1) [159] | **<0.001** |
| **Both Lungs, High opacity percentage** | 12 (9, 15) [431] | 11 (9, 14) [272] | 14 (11, 17) [159] | **<0.001** |
| **Lobe involvement** | .. | .. | .. | .. |
| **Number of involved lobes** | .. | .. | .. | 0.650 |
| **1** | 2/432 (0.5%) | 2/272 (0.7%) | 0/160 (0%) | .. |
| **2** | 0/432 (0%) | 0/272 (0%) | 0/160 (0%) | .. |
| **3** | 5/432 (1.1%) | 3/272 (1.1%) | 2/160 (1.3%) | .. |
| **4** | 19/432 (4.4%) | 14/272 (5.2%) | 5/160 (3.1%) | .. |
| **5** | 406/432 (94%) | 253/272 (93%) | 153/160 (95.6%) | .. |
| **Left Upper Lobe Opacity score** | 2 (1, 3) [431] | 2 (1, 3) [272] | 3 (2, 3) [159] | **<0.001** |
| **Left Upper Lobe Opacity score** | .. | .. | .. | **<0.001** |
| **0** | 5/431 (1.2%) | 5/272 (1.8%) | 0/159 (0%) | .. |
| **1** | 108/431 (25.1%) | 81/272 (29.8%) | 27/159 (17%) | .. |
| **2** | 130/431 (30.2%) | 90/272 (33.1%) | 40/159 (25.2%) | .. |
| **3** | 136/431 (31.6%) | 74/272 (27.2%) | 62/159 (39%) | .. |
| **4** | 52/431 (12.1%) | 22/272 (8.1%) | 30/159 (18.9%) | .. |
| **Left Lower Lobe, Opacity score** | 3 (2, 4) [431] | 3 (2, 3) [272] | 3 (2, 4) [159] | **<0.005** |
| **Left Lower Lobe, Opacity score** | .. | .. | .. | 0.055 |
| **0** | 4/431 (0.9%) | 3/272 (1.1%) | 1/159 (0.6%) | .. |
| **1** | 53/431 (12.3%) | 41/272 (15.1%) | 12/159 (7.5%) | .. |
| **2** | 107/431 (24.8%) | 70/272 (25.7%) | 37/159 (23.3%) | .. |
| **3** | 146/431 (33.9%) | 92/272 (33.8%) | 54/159 (34%) | .. |
| **4** | 121/431 (28.1%) | 66/272 (24.3%) | 55/159 (34.6%) | .. |
| **Right Upper Lobe, Opacity score** | 2 (1, 3) [432] | 2 (1, 3) [272] | 3 (2, 4) [160] | **<0.001** |
| **Right Upper Lobe, Opacity score** | .. | .. | .. | **<0.001** |
| **0** | 8/432 (1.9%) | 6/272 (2.2%) | 2/160 (1.3%) | .. |
| **1** | 105/432 (24.3%) | 85/272 (31.3%) | 20/160 (12.5%) | .. |
| **2** | 111/432 (25.7%) | 69/272 (25.4%) | 42/160 (26.3%) | .. |
| **3** | 125/432 (28.9%) | 74/272 (27.2%) | 51/160 (31.9%) | .. |
| **4** | 83/432 (19.2%) | 38/272 (14%) | 45/160 (28.1%) | .. |
| **Right Middle Lobe, Opacity score** | 2 (1, 3) [432] | 2 (1, 3) [272] | 2 (1, 3) [160] | **<0.001** |
| **Right Middle Lobe, Opacity score** | .. | .. | .. | **<0.001** |
| **0** | 18/432 (4.2%) | 14/272 (5.1%) | 4/160 (2.5%) | .. |
| **1** | 157/432 (36.3%) | 117/272 (43%) | 40/160 (25%) | .. |
| **2** | 119/432 (27.5%) | 72/272 (26.5%) | 47/160 (29.4%) | .. |
| **3** | 101/432 (23.4%) | 53/272 (19.5%) | 48/160 (30%) | .. |
| **4** | 37/432 (8.6%) | 16/272 (5.9%) | 21/160 (13.1%) | .. |
| **Right Lower Lobe, Opacity score** | 3 (2, 4) [432] | 3 (2, 4) [272] | 3 (3, 4) [160] | **<0.001** |
| **Right Lower, Lobe Opacity score** | .. | .. | .. | **<0.008** |
| **0** | 0/432 (0%) | 0/272 (0%) | 0/160 (0%) | .. |
| **1** | 55/432 (12.7%) | 43/272 (15.8%) | 12/160 (7.5%) | .. |
| **2** | 80/432 (18.5%) | 54/272 (19.9%) | 26/160 (16.3%) | .. |
| **3** | 163/432 (37.7%) | 104/272 (38.2%) | 59/160 (36.9%) | .. |
| **4** | 134/432 (31%) | 71/272 (26.1%) | 63/160 (39.4%) | .. |
| **Right Lung, Opacity score** | 7 (5, 9) [432] | 7 (5, 9) [272] | 8 (7, 10) [160] | **<0.001** |
| **Left Lung, Opacity score** | 5 (4, 7) [431] | 5 (3, 6) [272] | 6 (4, 7) [159] | **<0.001** |

Data are expressed as n (%), [N0: Number of patients with available data in case of missing data], n/N0, or median (IQR), unless stated otherwise.

# eFigure 2. Visual quantification of lung lesions identified at baseline in the fibrotic changes group according to time interval between CT acquisition and ICU admission

#
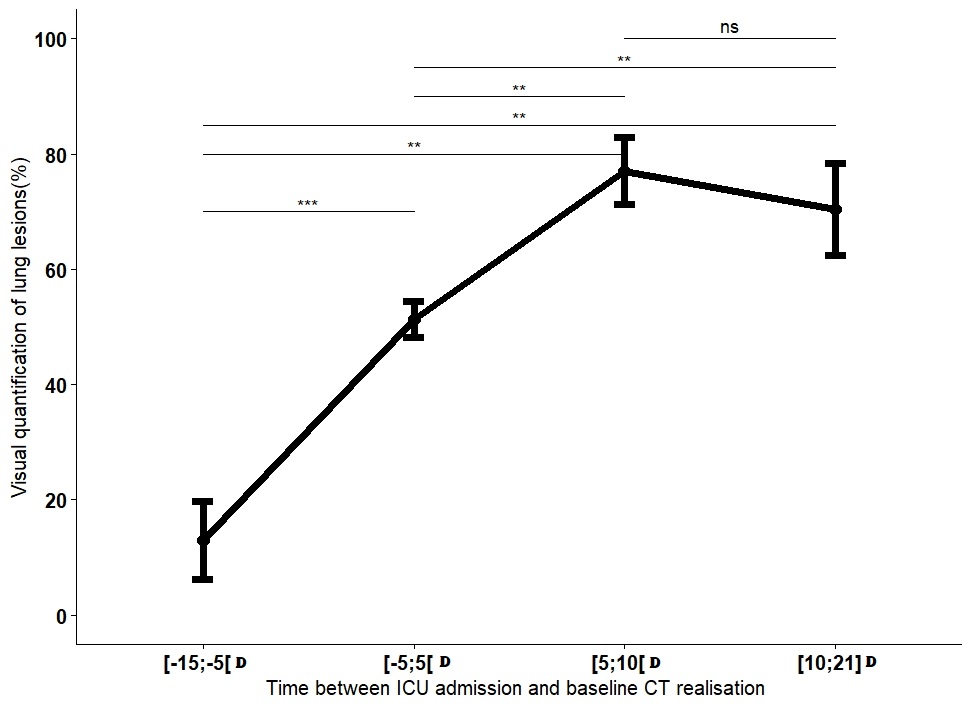


# eTable 3. Primary analysis: results of the multivariate analysis (full model) for predictive factors of fibrotic changes 6 months after ICU discharge

|  | **Full model** | | **Final model** | |
| --- | --- | --- | --- | --- |
|  | **aOR (95%-CI)** | **P-value** | **aOR (95%-CI)** | **P-value** |
| **Demographic and ICU Data** | .. | .. | .. | .. |
| **Age** | 1.04 (1.02-1.07) | 0.001 | 1.04 (1.02-1.07) | **0.001** |
| **Sex (Male)** | 1.63 (0.95-2.82) | 0.079 | 1.58 (0.93-2.71) | 0.092 |
| **BMI** | .. | .. | .. | .. |
| **<30** | **ref** | .. | **ref** | .. |
| **30-<40** | 0.57 (0.34-0.95) | 0.031 | 0.55 (0.33-0.90) | **0.018** |
| **≥40** | 0.24 (0.06-0.74) | 0.021 | 0.22 (0.06-0.67) | **0.013** |
| **Charlson comorbidity score** | .. | .. | .. | .. |
| **0** | **ref** | .. | **ref** | .. |
| **≥1** | 1.85 (1.14-3.04) | 0.014 | 1.72 (1.07-2.77) | **0.025** |
| **ICU related data** | .. | .. | .. | .. |
| **SAPSII** | 0.99 (0.97-1.02) | 0.812 | .. | .. |
| **SOFA** | 0.93 (0.83-1.03) | 0.151 | .. | .. |
| **ICU acquired pneumonia (Yes)** | 1.46 (0.82-2.61) | 0.193 | .. | .. |
| **Antiviral therapies (yes)** | 1.70 (0.96-3.03) | 0.069 | .. | .. |
| **Most invasive respiratory support** | .. | .. | .. | .. |
| **HFOT** | **ref** | .. | **ref** |  |
| **IMV** | 4.03 (2.02-8.24) | <0.001 | 4.22 (2.41-7.34) | **<0.001** |
| **NIV** | 2.46 (0.94-6.19) | 0.058 | 2.54 (0.99-6.28) | 0.047 |
| **Baseline chest CT data** | .. | .. | .. | .. |
| **Number of early fibrotic signs on baseline CT** | .. | .. | .. | .. |
| **0** | **ref** | .. | **ref** | .. |
| **1** | 1.03 (0.55-1.90) | 0.925 | 1.05 (0.57-1.90) | 0.883 |
| **2** | 1.64 (0.79-3.43) | 0.185 | 1.67 (0.81-3.48) | 0.168 |
| **≥3** | 3.75 (1.47-10.12) | 0.006 | 3.70 (1.49-9.86) | **0.006** |
| **Crazy Paving (Yes)** | 1.37 (0.80-2.35) | 0.249 | .. | .. |
| **Visual evaluation of lung involvement** | 1.03 (1.02-1.04) | <0.001 | 1.03 (1.02-1.04) | **<0.001** |

ICU, intensive care unit; aOR, adjusted odds ratio; CI, confidence interval; BMI, body mass index; SPASII, Simplified Acute Physiology Score II; SOFA, Sequential Organ Failure Assessment score; HFOT, high flow oxygen therapy; IMV, invasive mechanical ventilation; NIV, non-invasive mechanical ventilation; CT, computed tomography

**eFigure 3. ROC Curves for evaluating the final model accuracy in predicting fibrotic changes 6 months after ICU discharge, calibration plot and ROC curve (AUC) for the final logistic regression model**


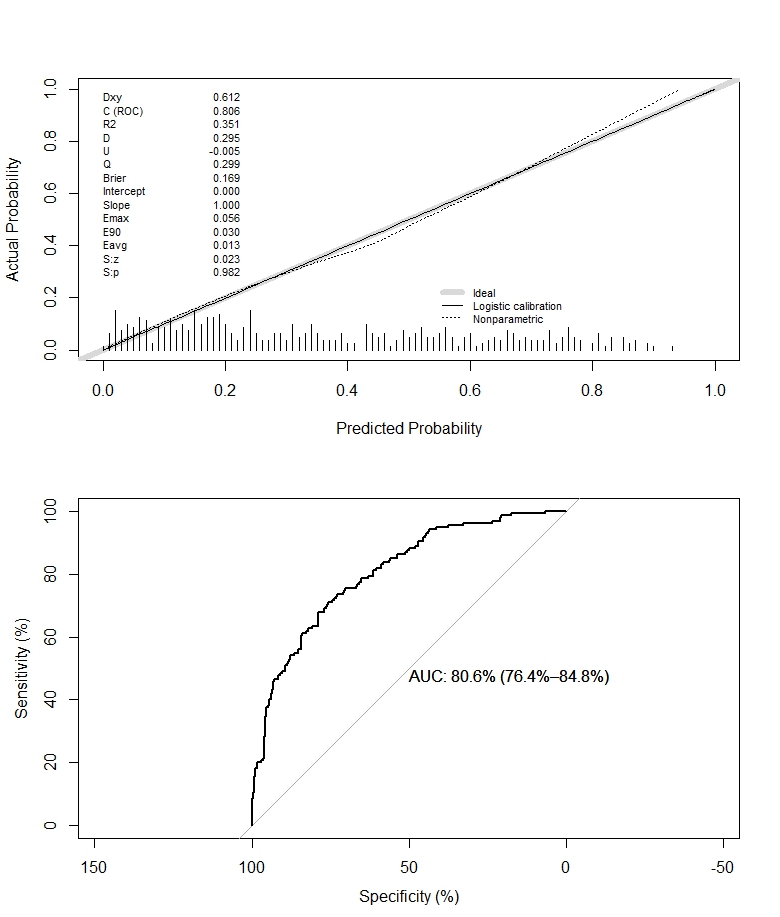
 **
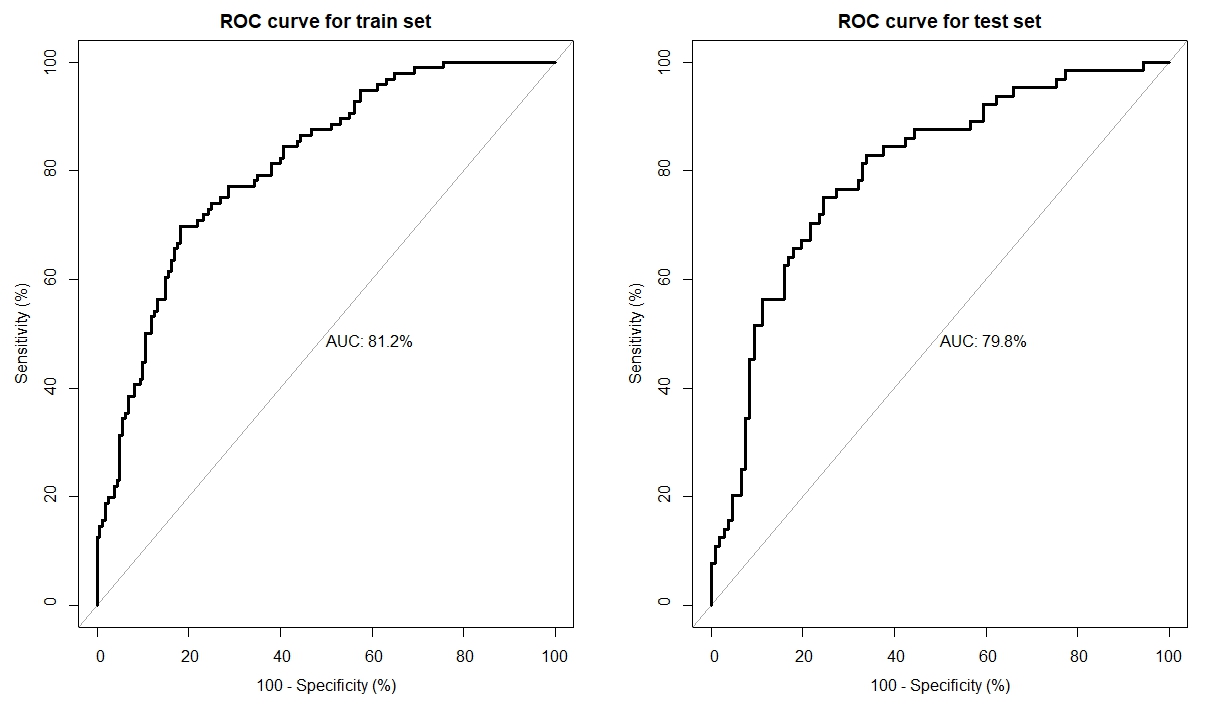
**

# eTable 4. Descriptive comparison of final model variables between training and test sets

|  | **Test Data** | **Train data** |  |
| --- | --- | --- | --- |
|  | **N = 87** | **N = 353** | **p-value** |
| **Fibrotic Changes** | 32 (37%) | 130 (37%) | 0.994 |
| **Age** | 61.89(11.60) | 63.57(10.89) | 0.294 |
| **Sex** | .. | .. | 0.696 |
| Female | 28 (32%) | 106 (30%) | .. |
| Male | 59 (68%) | 247 (70%) | .. |
| **BMI** | .. | .. | 0.250 |
| <30 | 54 (62%) | 194 (56%) | .. |
| 30-<40 | 25 (29%) | 132 (38%) | .. |
| **≥**40 | 8 (9.2%) | 23 (6.6%) | .. |
| Missing | 0 | 4 | .. |
| **Charlson comorbidity score** | .. | .. | 0.108 |
| 0 | 42 (48%) | 203 (58%) | .. |
| **≥**1 | 45 (52%) | 148 (42%) | .. |
| Missing | 0 | 2 | .. |
| **Number of fibrotic signs on baseline CT** | .. | .. | 0.154 |
| 0 | 61 (70%) | 223 (63%) | .. |
| 1 | 18 (21%) | 61 (17%) | .. |
| 2 | 5 (5.7%) | 44 (12%) | .. |
| **≥**3 | 3 (3.4%) | 25 (7.1%) | .. |
| **Most invasive respiratory support** | .. | .. | 0.808 |
| HFOT | 28 (32%) | 113 (32%) | .. |
| IMV | 53 (61%) | 208 (59%) | .. |
| NIV | 6 (6.9%) | 32 (9.1%) | .. |
| **Visual quantification of lung lesions on baseline CT** | 46.25(28.12,61.88) | 44.38(27.50,60) | 0.520 |

BMI, body mass index; CT, computed tomography; HFOT, high flow oxygen therapy; ICU, intensive care unit; IMV, invasive mechanical ventilation; NIV, non-invasive mechanical ventilation

# eFigure 4. Global visual quantification of lung lesions identified on follow-up CT in the entire cohort according to time interval between ICU discharge and follow-up CT

#
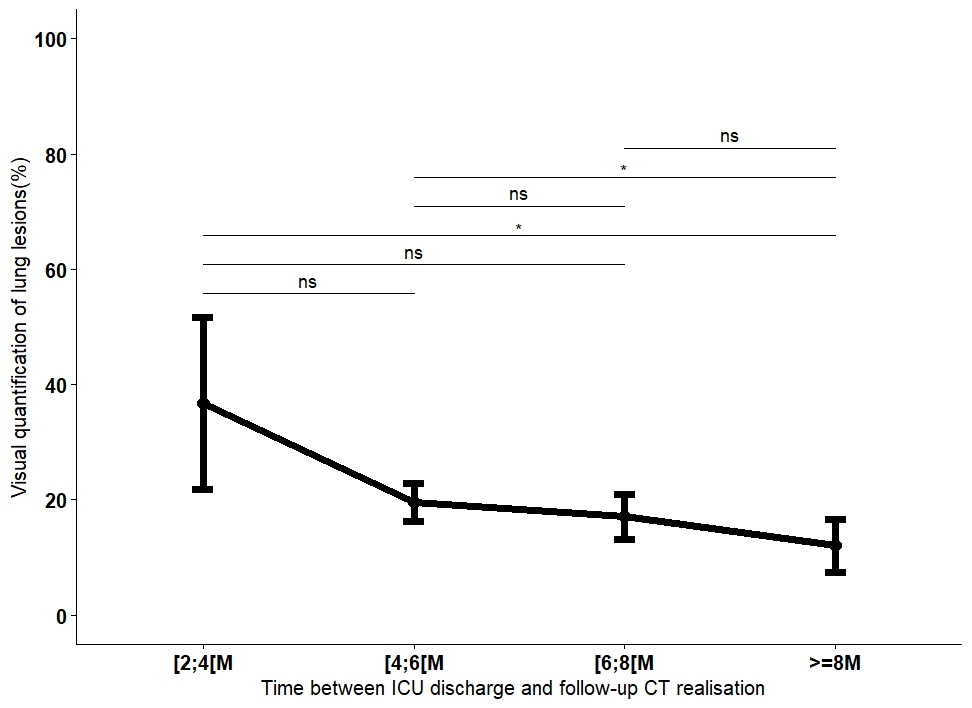


*p<0.05; ns: not statistically significant

# eFigure 5. Prevalence of fibrotic changes on follow-up according to time interval between ICU discharge and follow-up CT

**
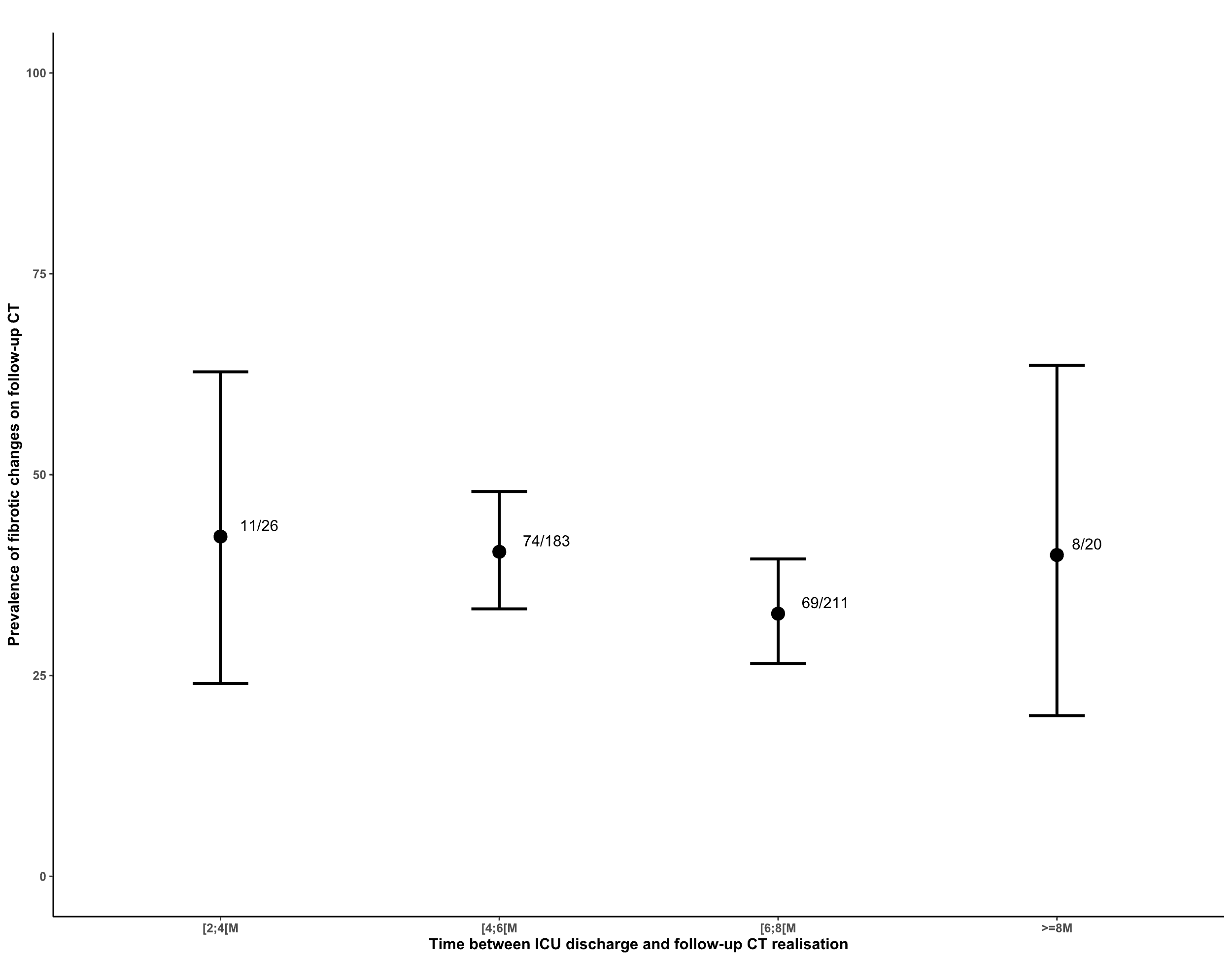
**

p>0.05 overall and between pairs

# eTable 5. Respiratory outcomes 6 months after ICU discharge according to fibrotic changes

|  | **All patients, N=440** | **No Fibrotic Changes, N=278** | **Fibrotic Changes, N=162** | **P-value** |
| --- | --- | --- | --- | --- |
| **Dyspnea, mMRC scale ≥2** | 98/393 (24.9%) | 53/247 (21.5%) | 45/146 (30.8%) | **0.038** |
| **Pulmonary function tests** | .. | .. | .. | .. |
| FEV1, % | 100 (87, 112) [425] | 100 (88, 113.5) [267] | 99 (82, 109) [158] | 0.227 |
| FVC, mean (SD), % | 96.1 (19.9) [425] | 98.9 (18.7) [269] | 91.4 (21.1) [156] | **<0.001** |
| FEV1/FVC, % | 82 (77.2, 86.8) [425] | 81.6 (76.8, 86.4) [270] | 82.9 (77.7, 87.7) [155] | 0.212 |
| TLC, mean (SD), % | 88.5 (15.4) [406] | 92.1 (14.3) [257] | 82.2 (15.2) [149] | **<0.001** |
| RV, % | 85 (69, 100.7) [402] | 81.6 (76.3, 104) [254] | 77 (60, 92) [148] | **<0.001** |
| DL_CO_, % | 75 (62, 85) [405] | 78 (69, 89) [257] | 68 (56, 78) [148] | **<0.001** |
| K_CO,_ % | 92 (80, 101) [366] | 93 (83, 103) [233] | 89 (77, 99) [133] | **0.016** |
| **6-minute walk test** | .. | .. | .. | .. |
| Distance, m | 450 (360, 521) [415] | 460 (376.5, 522.5) [263] | 430.5 (344, 520.5) [152] | 0.082 |
| SpO_2_ before test, % | 97 (96, 98) [419] | 97 (96, 98) [269] | 97 (95, 98) [150] | **0.029** |
| SpO_2_ at end of the test, % | 95 (92.5, 97) [403] | 95 (93.5, 97) [255] | 94 (91, 96) [148] | **<0.001** |
| Dyspnea (Borg scale) before test | 0 (0, 1) [396] | 0 (0, 1) [250] | 0 (0, 1) [146] | 0.256 |
| Dyspnea (Borg scale) after test | 2 (1, 4) [397] | 2 (1, 4) [252] | 3 (1, 4) [145] | 0.286 |
| **Respiratory quality of life** | .. | .. | .. | .. |
| VSRQ scale | 57 (42, 69) [409] | 57 (42, 70) [257] | 56.5 (43, 66) [152] | 0.523 |

Data are expressed as n (%), [N0: Number of patients with available data in case of missing data], n/N0, or median (IQR), unless stated.

ICU, intensive care unit; mMRC, the modified Medical Research Council dyspnea scale; FEV_1_, forced expiratory volume in 1 second; FVC, forced vital capacity; TLC, total lung capacity; RV, residual volume; DL_CO_, diffusion capacity of the lung for carbon monoxide; K_CO_, carbon monoxide transfer coefficient; SpO2, peripheral capillary oxygen saturation; VSRQ, Visual Simplified Respiratory Questionnaire

# eTable 6. Multivariable median quantile regression analysis: effect of fibrotic changes and emphysema during follow-up on 6MWT walking

|  | **Beta (m)** | **CI_inf**  **(m)** | **CI_sup**  **(m)** | **p_value** |
| --- | --- | --- | --- | --- |
| **Fibrotic changes** |  |  |  |  |
| No | - | - | - |  |
| Yes | -30 | -65 | 4.8 | 0.092 |
| **Emphysema** |  |  |  |  |
| No | - | - | - |  |
| Yes | -17 | -46 | 12 | 0.301 |
|  |  |  |  |  |

6MWT, 6-minute walk test; CI, confidence interval margin; inf, inferior; sup, superior

*Multivariable median quantile regression analysis: the presence of fibrotic changes at 6 months was associated with an approximate 30-meter reduction in 6-minute walk distance (ß = –30 m, 95% CI: approximately –65 to 4.8), showing a trend toward statistical significance (p = 0.092) and suggesting a potential moderate impact of fibrotic sequelae on functional capacity. In contrast, the presence of emphysema was not significantly associated with 6MWT performance (ß = –17 m, 95% CI: approximately –46 to 12; p = 0.301). These findings suggest that the observed reduction in exercise capacity is more likely attributable to fibrotic changes than to emphysematous lesions, although the effect of fibrosis requires confirmation in larger cohorts or through alternative analytic approaches.*

# eFigure 6. Quality of life according to the 8 subscales of the SF-36 questionnaire and fibrotic changes status 6 months after ICU discharge

**
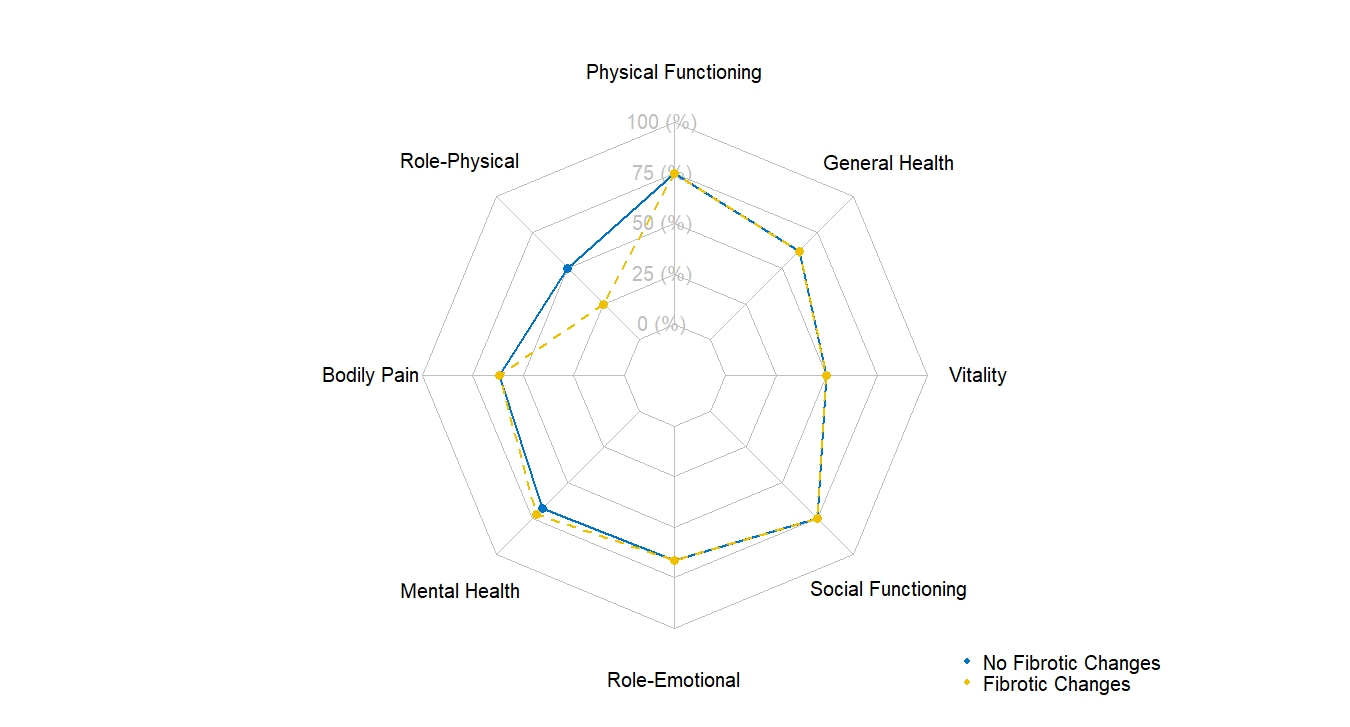
**

eFigure 7. SF-36 General health assessment, physical and mental summary scores at 6 months after ICU discharge according to fibrotic changes
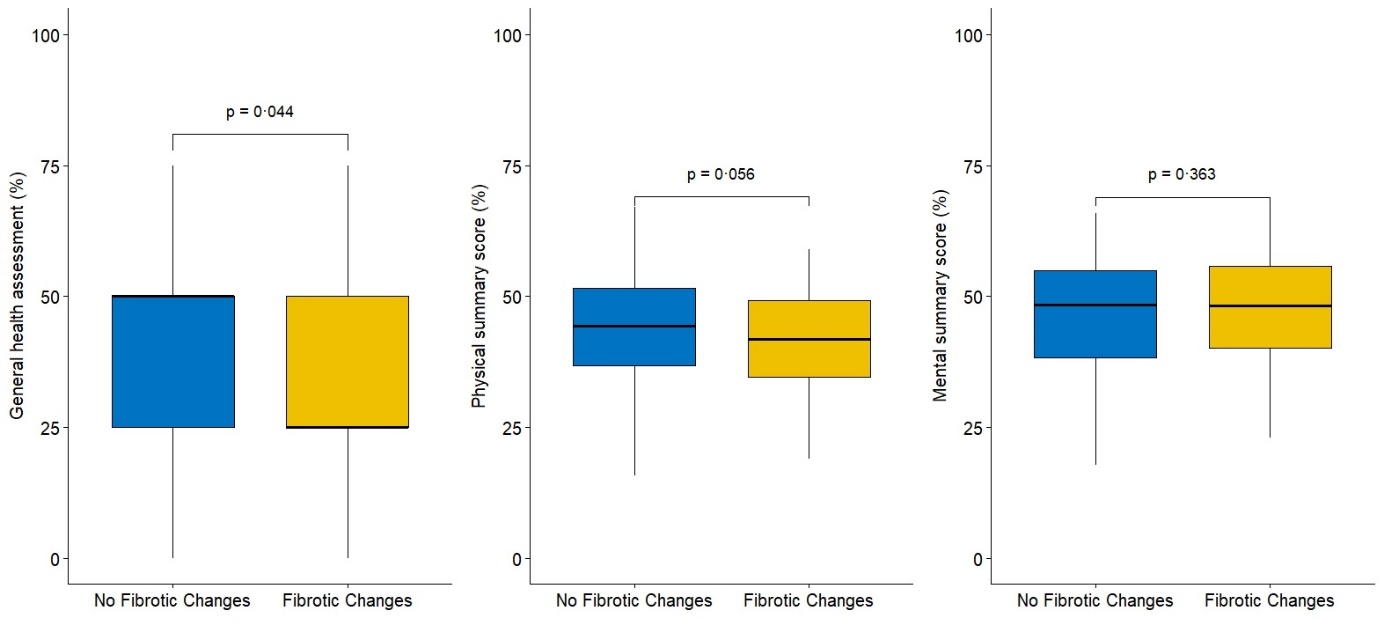
**Supplementary Table 1. Sensitivity analysis comparing study population with the subjects excluded due to lack of follow-up Chest CT** (not included in the revised manuscript)

|  | **Excluded Patients (no follow-up CT) N = 75** | **Included Patients N = 440** | **p-value** |
| --- | --- | --- | --- |
| **Age** | 61 (52, 71) [75] | 65 (56, 71) [440] | 0.060 |
| **Sex** |  |  | 0.51 |
| Female | 20/75 (27%) | 134/440 (30%) |  |
| Male | 55/75 (73%) | 306/440 (70%) |  |
| **BMI** |  |  | 0.27 |
| <30 | 33/70 (47%) | 248/436 (57%) |  |
| 30-<40 | 32/70 (46%) | 157/436 (36%) |  |
| >=40 | 5/70 (7.1%) | 31/436 (7.1%) |  |
| Missing | 5 | 4 |  |
| **Charlson Comorbidity score** |  |  | 0.35 |
| 0 | 36/72 (50%) | 245/438 (56%) |  |
| >=1 | 36/72 (50%) | 193/438 (44%) |  |
| Missing | 3 | 2 |  |
| **Number of fibrotic signs on base line CT** |  |  | 0.80 |
| 0 | 18/26 (69%) | 284/440 (65%) |  |
| 1 | 3/26 (12%) | 79/440 (18%) |  |
| 2 | 4/26 (15%) | 49/440 (11%) |  |
| >=3 | 1/26 (3.8%) | 28/440 (6.4%) |  |
| Missing | 49 | 0 |  |
| **Most invasive respiratory support** |  |  | 0.79 |
| HFOT | 20/71 (28%) | 141/440 (32%) |  |
| IMV | 44/71 (62%) | 261/440 (59%) |  |
| NIV | 7/71 (9.9%) | 38/440 (8.6%) |  |
| Missing | 4 | 0 |  |
| **Visual quantification of lung lesion on baseline CT** | 48 (26, 61) [25] | 45 (28, 61) [440] | 0.74 |
| Missing | 50 | 0 |  |
| **IGS II Score** | 31 (24, 41) [72] | 34 (28, 43) [438] | 0.068 |
| Missing | 3 | 2 |  |
| **SOFA Score** | 4 (3, 6) [72] | 4 (3, 6) [440] | 0.17 |
| Missing | 3 | 0 |  |

|  | **Overall**  N = 440 | **No FC at follow up**  N = 278 | **FC at follow up**  N = 162 | **p-value** |
| --- | --- | --- | --- | --- |
|  |  |  |  |  |
| **Bronchectasis or architectural distorsion** | **161 (37%)** | **0 (0%)** | **161 (99%)** | <0.001 |
| **Number of signs presents (bronchiectasis or architectural distorsion)** |  |  |  | <0.001 |
| 0 | 279 (63%) | 278 (100%) | 1 (0.6%) |  |
| Only one | 66 (15%) | 0 (0%) | 66 (40.7%) |  |
| Both signs | 95 (22%) | 0 (0%) | 95 (58.6%) |  |

**Supplementary Table 2. Prevalence of traction bronchiectasis and architectural distortion combined (not included in the revised manuscript)**
